# Dual destructive and protective roles of adaptive immunity in neurodegenerative disorders

**DOI:** 10.1186/2047-9158-3-25

**Published:** 2014-11-13

**Authors:** Kristi M Anderson, Katherine E Olson, Katherine A Estes, Ken Flanagan, Howard E Gendelman, R Lee Mosley

**Affiliations:** Department of Pharmacology and Experimental Neuroscience, Center for Neurodegenerative Disorders, The University of Nebraska Medical Center, Omaha, NE 68198 USA; Prothena Biosciences, South San Francisco, 650 Gateway Boulevard, CA 94080 USA

**Keywords:** Neurodegeneration, Neuroprotection, Migration, MPTP, MCAM, Regulatory T cell, Effector T cell, Neuroinflammation

## Abstract

Inappropriate T cell responses in the central nervous system (CNS) affect the pathogenesis of a broad range of neuroinflammatory and neurodegenerative disorders that include, but are not limited to, multiple sclerosis, amyotrophic lateral sclerosis, Alzheimer’s disease and Parkinson’s disease. On the one hand immune responses can exacerbate neurotoxic responses; while on the other hand, they can lead to neuroprotective outcomes. The temporal and spatial mechanisms by which these immune responses occur and are regulated in the setting of active disease have gained significant recent attention. Spatially, immune responses that affect neurodegeneration may occur within or outside the CNS. Migration of antigen-specific CD4+ T cells from the periphery to the CNS and consequent immune cell interactions with resident glial cells affect neuroinflammation and neuronal survival. The destructive or protective mechanisms of these interactions are linked to the relative numerical and functional dominance of effector or regulatory T cells. Temporally, immune responses at disease onset or during progression may exhibit a differential balance of immune responses in the periphery and within the CNS. Immune responses with predominate T cell subtypes may differentially manifest migratory, regulatory and effector functions when triggered by endogenous misfolded and aggregated proteins and cell-specific stimuli. The final result is altered glial and neuronal behaviors that influence the disease course. Thus, discovery of neurodestructive and neuroprotective immune mechanisms will permit potential new therapeutic pathways that affect neuronal survival and slow disease progression.

**Electronic supplementary material:**

The online version of this article (doi:10.1186/2047-9158-3-25) contains supplementary material, which is available to authorized users.

## Introduction

The pathobiology of neurodegenerative disorders has proven complex and multi-faceted. One commonality between the disorders is the involvement of innate and adaptive immune responses in the central nervous system (CNS)[1]. The interplay between peripheral and resident CNS immunity can affect neuroinflammatory responses and exacerbate neurodegeneration[2]. Previously, the CNS was believed to be immune privileged and deprived of infiltration of peripheral immune cells[3, 4]. This has since been disproven on several grounds[4, 5]. *First*, the CNS is composed of a unique blood brain barrier (BBB) that affords limits and control over the infiltration of peripheral immune cells[4]. Control of immune cell infiltration into the CNS is regulated through unique cell adhesion molecules (CAMs) and CAM ligands on BBB endothelial cells. *Second*, during neuroinflammatory responses, the expression of these CAMs can be upregulated, allowing a greater number of adaptive immune cells to cross the BBB and interact with resident immune cells in the brain[6–11]. *Third*, T cells, such as regulatory T cells, can elicit a neuroprotective response, thus complete ablation of T cell infiltration to the CNS could be detrimental to the host[12–14]. Therapies that competitively interact with CAMs and CAM ligands can modulate the extravasation of peripheral immune cells into the CNS. Those pharmaceutical agents that more selectively inhibit migration of pro-inflammatory or deleterious T cells, but allow anti-inflammatory or regulatory T cell migration could evoke beneficial and neuroprotective responses. To these ends, the current review article aims to describe the role of immune cells in neurodegenerative disorders, migration of these immune cells from the periphery into the CNS, the interactions between innate and adaptive immune responses, and pharmacologic immune modulators in neuroprotection. Lastly, primary data will be discussed that support findings linking the migration of specific T cell subsets in a disease-relevant model.

## T cells and adaptive immunity

T cells represent one arm of the adaptive immune system which is responsible for mounting a response against foreign antigen. T cells are derived from bone marrow lymphocyte progenitors that mature and are educated within the thymus; whence they migrate to the periphery and reside throughout the tissues of the body, but mainly in spleen, lymph nodes, and peripheral circulation. T cells, acting as effector cells, provide the impetus and signals for directing the cellular and antibody responses necessary to clear foreign pathogens and antigens, but also, as regulatory T cells (Tregs), endow immunological tolerance to the individual and actively restrains one’s immune system from recognizing itself as foreign.

### Adaptive immunity

Both innate and adaptive immune responses are important for mounting the body’s defense against a pathogen or foreign microorganism. The innate response is the first line of defense, which is relatively rapid, recognizes a broad spectrum of antigen patterns, does not require immune memory, and is characterized by phagocytic activity mediated by resident mononuclear phagocytes such as macrophages, dendritic cells, and microglia. The adaptive immune response requires substantially greater time to develop, produce and utilize immunological memory, and affords narrow specificity for antigens based on lymphoid receptors for antigen expressed by T cells [T cell receptors (TCRs)] and B cells [immunoglobulins (Igs)]. The main function of the adaptive immune system is to recognize foreign invaders, destroy foreign microorganisms or pathogens, and relieve pathogen-associated toxicities. However, to initially mount an immune response, the innate arm of the immune system must first be activated by recognition via broadly-specific pattern recognition receptors (PRRs) which recognize microorganism-associated molecular patterns (MAMPs) or damage-associated molecular patterns (DAMPs). In an innate response, antigens recognized by PRRs of mononuclear phagocytes are engulfed, digested, and processed to bind with molecules of the major histocompatibility complex (MHC). Upon maturation, mononuclear phagocytes acquire an antigen presenting capability whereby processed and MHC-bound antigen is expressed on the cell surface in a configuration necessary for T cells to recognize the antigen via the TCR. Unlike Igs which recognize cellular- or non-cellular-bound antigen, the TCR only recognizes antigen presented by MHC molecules on antigen presenting cells (APCs). APCs also express co-stimulatory molecules such as CD80, CD86, CD70, CD40, and CD200 that are necessary to generate an effective, robust, and specific immune response. As APCs, these mononuclear phagocytes bridge the innate and adaptive arms of the immune system by providing the antigen and co-stimulation necessary for naïve T cells to become activated. Additionally, APCs also deliver cytokine signals, such as IL-12, IL-4, IL-6, and TGF-β that direct naïve T cells to follow a program of polarized differentiation and transforms them into activated T cells with specific effector functions (see below, T cell subsets). Once activated, T cells proliferate and undergo clonal expansion to increase their cell number and potential to challenge invading pathogens. One mechanism by which effector T cells expand in the presence of antigen is to secrete pro-growth cytokines to the surrounding environment. For instance, activated T cells produce and secrete IL-2 cytokine that binds their own IL-2R to enhance proliferation in an autocrine fashion, as well as enhance proliferation of surrounding T cells in a paracrine fashion[15]. In addition, to achieve as efficient effector function as possible, activated T cells migrate to areas of infection and inflammation to interact with other immune cells, such as macrophages or microglia, and bestow collaborative effector functions to rid the host of a foreign assault. Thus, T cells are important for the general cellular-mediated response of the adaptive immune arm.

### T cell subsets

T cells are generally characterized by expression of the TCR-CD3 complex on the cell surface. While the TCR recognizes presented antigen, it does not possess the cytosolic machinery necessary for successful signal transduction; however, the CD3 complex serves as the signaling mechanism that bridges the antigen recognition and conveyance of signal for effector function. T cells are subdivided into two major lineages based on the expression of either CD4 or CD8 on the cell surface. CD4+ T cells are considered helper T (Th) cells. With the TCR recognizing the antigen, the CD4 molecule acts as a co-receptor and binds the MHC II molecule that presents the antigen from the APC. Depending on the cytokines generated by the APC, activated CD4+ T cells develop into specific effector T cell (Teff) subsets that include type-1, -2, -17 or -9 Th cells designated as Th1, Th2, Th17, or Th9 cells, respectively, as well as Tregs. Th subsets secrete a variety of cytokines that act as either pro- or anti-inflammatory mediators. Classically, Th1 cells are important for mounting immune responses against intracellular pathogens and are characterized by secretion of predominately IFN-γ, TNF-α, and IL-2[16, 17]. IFN-γ from Th1 cells enhances macrophage activation necessary for immunity to pathogens that cause listeriosis, tuberculosis, salmonellosis, and leishmaniasis. In addition, Th1 cells produce IL-2 and IL-21 that promote and maintain antigen-specific CD8+ cytotoxic T lymphocytes (CTLs) that lyse virus-infected cells. CTLs are primarily responsible for ridding the host of intracellular pathogens and virus-infected cells. CTLs recognize antigen presented by MHC class I, which is expressed by most cells in the body. When activated and expanded, CTL effectors produce perforins and granzymes to induce cell-mediated cytolysis[18]. On the other hand, Th2 cells preferentially generate help for antibody-mediated responses, mainly through the secretion of IL-4, IL-10, IL-13, and IL-5, which act on B cells for antibody production. Because the prototypical cytokines from Th1 and Th2 cells regulate the expression of each other’s master-controlling transcription factors, this preferentially selects T cells to differentiate into either Th1 or Th2 effector cell types in response to pathogens[16]. Th17 cells are a more recently discovered subset of Teffs. They predominantly secrete IL-17, as well as TNF-α, and are thought to be important for protection against extracellular infections, and as such, yield a particularly heavy armament as a destructive Teff type due primarily to the pro-inflammatory milieu secreted in response to extracellular pathogens[19]. In addition to the Th17 effectors, another subset expresses IL-9 and is thought also to be important in resolution of extracellular infections. The Th9 subset has been implicated in promoting the migration of Th17 cells to the CNS[20] and potentiation of Th17 effectors via the ability to increase Th17-produced IL-17. Moreover, recent evidence indicates that other T cell types such as Tregs, Th1, and Th17 also can secrete IL-9 with pleiotropic effects that may ultimately alter the predominant proinflammatory response[21]. Th22 cells are a separate lineage of CD4+ Teffs that primarily secrete proinflammatory cytokines such as IL-22, IL-13 and TNF-α, and express the skin homing-associated chemokine receptors CCR4, CCR6 and CCR10[22]. Th22 cells differentiate from naïve T cells in the presence of IL-6 and TNF-α under control of the transcription factors aryl hydrocarbon receptor and GATA3. These Teffs are recruited to the skin and thought to be involved in microbial immunity and tissue repair and remodeling. Skin disorders such as psoriasis, eczema, and contact dermatitis may be due to dysregulation of Th22 migration or function. Each of these T cell subsets plays crucial, yet independent roles in mounting a robust and effective adaptive immune response. As these effector cells provide potent weapons toward immunity to foreign invaders, they also serve equally as potential liabilities due to increased pro-inflammatory cytokines, antibody-mediated cytotoxicity to cells, or hyperactivation of innate immune cells. Thus, regulation of the Teffs is necessary to prevent untoward pathological sequelae.

CD4+CD25+ Tregs provide that regulation, ostensibly via expression and control of a master regulatory transcription factor, forkhead box 3 (FoxP3). Tregs are a subset of T cells that regulate and suppress the activities of Teffs and myeloid lineage cells such as dendritic cells, microglia, and macrophages that comprise innate immunity. Tregs can be further subdivided into natural Tregs (nTregs) that are derived from the thymus and induced Tregs (iTregs) that arise from naïve T cells in the periphery[23]. Moreover, a primary function of Tregs is the maintenance of immunological tolerance to self and thus, inhibition of initial auto-immune responses and suppression of auto-reactive T cells that may arise in the peripheral tissues. Individuals that do not produce functional nTregs due to mutated *FOXP3*, develop immunodysregulation polyendocrinopathy enteropathy X-linked (IPEX) syndrome, a systemic, multi-organ autoimmune disorder[24]. Treg-mediated regulatory functions are achieved at many levels. First, Tregs inhibit initiation of immune responses by diminishing antigen processing and antigen presentation by MHC molecules as well as regulating 2nd and 3rd signals from APCs. Second, Tregs secrete anti-inflammatory cytokines such as IL-10, IL-35, and TGF-β that suppress activated mononuclear phagocytes and Teffs. Unlike the Th2 subset, which also elicits anti-inflammatory and protective effects, Tregs do not traditionally interact with B cells to elicit a response.

## T cells in neurodegenerative disorders

The interplay between the innate and adaptive arms of the immune system is essential to the relationship between neuroinflammation, neuroprotection, and neurodegeneration. While neuroinflammation and neurodegeneration are associated with the pathobiology of neurodegenerative diseases, they are also responsible for the overall neuroprotective homeostasis of the host CNS in infectious or neoplastic disease surveillance. Similarities between multiple neurological disorders have provided common mechanisms of immune interactions that lead to protective or destructive effects within the CNS and peripheral nervous system (PNS). Although neuroinflammation and T cell interactions play a prominent role in disease progression, it should be noted that the immune response can vary from a very prominent primary T cell response, as in multiple sclerosis (MS), to seemingly less intense, though present, T cell response as in the cases of amyotrophic lateral sclerosis (ALS), Alzheimer’s disease (AD) and Parkinson’s disease (PD). However, it should also be noted that detrimental secondary inflammatory responses are observed in these neurodegenerative diseases, particularly in AD, PD and ALS, thus this commonality warrants further elaboration. Recent findings in human neurodegenerative disorders and in corresponding animal models have shown the involvement and putative mechanisms of T cells and subsequent secondary responses in disease initiation and progression.

### Multiple sclerosis

MS is a chronic, progressive demyelinating inflammatory disorder that is principally driven by T cells specific for self-antigens expressed in the myelin sheath[25, 26]. This notion is supported largely by data showing the presence of activated myelin-reactive T cells as well as CD4+ T cell infiltrates in MS patients affect the disease course[27–29]. As such, therapeutic modalities and clinical trial strategies have primarily targeted components of the immune system. While no modality has proven to be curative, and clinical trial outcomes varied extensively with little to no efficacy, some with beneficial effect, and others with devastating side effects, results have furthered our understanding on the pathogenesis of MS[30].

Evidence supports activated CD4+ myelin-reactive T cells as a driving force behind MS[29]. However, a compounding complexity of the disease arises from the finding that both healthy controls and MS patients have similar numbers of circulating T cells reactive to components of myelin[29]. Thus, the mere presence of self-reactive T cells is not sufficient evidence to explain the development of MS. Prior to the discovery of Th17 cells, MS was considered a purely Th1-mediated disease. However, recent studies lead to the view that MS is neither a purely Th1- nor Th17-mediated disease. As with EAE, both Teff subtypes are thought to participate in the pathology of MS, but with relative dominance of each Teff type playing a critical role in the progression of MS, affecting the temporal course and clinical variants[27]. For instance, T cells stained for expression of IL-17 are reported to be higher in early active plaques compared with chronic active or inactive plaques[31]. Similar *ex vivo* data showed that peripheral blood mononuclear cells (PBMCs) derived from MS patients taken within 2 years of diagnosis produced higher levels of IL-17 *in vitro* compared with those taken from patients with long-standing disease[32].

The frequencies of Tregs in both the blood and cerebral spinal fluid (CSF) of MS patients have been extensively investigated[33–36]. Interestingly, when brain tissue was examined from 16 untreated MS patients, no Tregs were found in 30% of the biopsies, and the number of FoxP3+ cells was generally low in the brain tissue[37] suggesting Tregs may not be capable of infiltrating the CNS in MS patients, and therefore, immune responses are un-regulated. While further studies showed no significant differences in the number of Tregs from the peripheral blood or CSF of MS patients compared to healthy controls, the functional capabilities of Tregs were impaired in patients suffering from MS[38].

The functional impairment of Tregs from MS patients could not be attributed to a higher activation status of Teffs, but rather seemed intrinsic to the Tregs themselves[38]. Indeed, experiments examining Treg functionality led by separate investigators found MS patients had lower mRNA and protein expression levels of the Treg transcription factor, FOXP3, when compared to healthy controls[38–40]. Venken *et al.* made similar findings in patients suffering from relapsing-remitting MS. However, FOXP3 expression and Treg functionality was normal during secondary progressive MS[40]. Whether Treg dysfunction in MS represents a general defect in the regulatory network of the immune system, and as such is a causative factor, remains to be elucidated[38].

Experimental autoimmune encephalomyelitis (EAE) has been the primary model of CNS autoimmune disease for over half a century[41]. The use of EAE has expanded the understanding of immune regulation of autoimmune disease. Furthermore, the EAE model affords evidence reaching beyond MS, providing mechanisms by which Teffs gain entry into the brain[6]. In adoptive transfer studies of EAE, researchers have shown that myelin-reactive T cells polarized to either a Th1 or Th17 phenotype are capable of initiating disease in recipient mice, but the histopathological outcome from the two T cell populations were distinct. In animals that received Th1 polarized cells, macrophages were more prominent, whereas Th17 recipient mice showed a more severe neutrophil infiltration[42]. This suggested that while both Th1 and Th17 cells play a role in de-myelination and disease progression, their mechanisms of destruction may be different. In addition to demonstrating different subsets of Teffs that elicit different pathological signs in EAE, studies also showed a temporal involvement of Th1 and Th17 in disease progression. Results demonstrated an early involvement of Th17 cells, with Th1 cells becoming predominant prior to disease resolution. Dardalhon and colleagues proposed this shift in polarization might be related to the natural course and recovery from an attack of EAE[43].

To address the role of CD25+ T cells in autoimmunity, Sakaguchi and colleagues demonstrated that nude mice reconstituted with CD4+ T cells depleted of the CD25+ subpopulation of cells developed spontaneous autoimmune disease[44]. Replenishment of the CD25+ cell population prevented this development of autoimmune diseases. This suggested the presence of a naturally arising subset of T cells that acted to limit the response to self-antigens[41]. Several more recent studies showed that Treg depletion prior to EAE induction increased the severity of the disease[41, 45–47] indicating that Tregs suppress expansion of autoreactive effector cells.

### Amyotrophic lateral sclerosis

ALS is a progressive neurodegenerative disease of unknown origin that primarily affects upper and lower motor neurons located at the ventral horn of the spinal cord, brain stem, and motor cortex[48]. These regions control voluntary muscle movement, leading to paralysis, respiratory failure, and ultimately, death with disease progression[49]. Although the etiology remains enigmatic, many factors and genetic mutations contribute to the pathobiology of the disease in some cases. In familial ALS (fALS), mutation of genes such as those that encode Cu/Zn superoxide dismutase (SOD-1) on chromosome 21 or alsin that encodes a ras GTPase have been linked to ALS patient populations[50, 51]. Currently over 20 different genes have been associated with fALS; however, the majority of ALS cases are sporadic and not linked to familial or genetic factors. As a contributing factor, neuroinflammation is thought to play a prominent role, and is supported by evidence of reactive microglia and astrocytes as well as infiltrating T cells found at affected sites and implicated in disease pathogenesis[52]. The interplay between nervous and immune systems results in an inflammatory response, which can be detrimental or protective depending on the disease state. Activated Teffs have the ability to penetrate the BBB and carry out their immune functions in the CNS[53], and inflammation has been thought to play a crucial role in the death of motor neurons[54], suggesting that perhaps an aberrant adaptive immune response is occurring.

Substantial numbers of infiltrating T cells and macrophages are found in the spinal cords of patients[55, 56]. The majority of these migrating T cells are described as CD8+ cytotoxic T cells with CD4+ T cells usually comprising a minority of lymphocytes. A considerable number of T cells are in close proximity to vessels near sites of neurodestruction[57], while little or no T cell infiltration is found in spinal cords of controls. An immunohistochemical study by Engelhardt *et al*. found both perivascular and intraparenchymal lymphocytic infiltrates in the post-mortem spinal cords of 18 ALS patients[6]. Virtually all lymphocytes were T cells with little B cell infiltration, suggesting a T cell specific mechanism of destruction. Mostly, activated CD4+ T cells were found near degenerating spinal tracts in ALS patients[58]. Apart from direct migration into affected areas such as the spinal cord, more immune aberrations have also been documented in the periphery. For instance, the frequencies of CD4+ T cells are increased in the peripheral blood of sporadic ALS patients as well as increased expression of antigen presenting molecules like HLA class II on APCs, suggesting systemic immune activation in those patients[59]. While these changes are found primarily in the periphery, those occurring in the CNS may be quite different. Indeed, Teff types found infiltrating the CNS were characteristic of Th17 and Th1, suggesting the involvement of IL-17, IFN-γ, TNF-α and IL-6 proinflammatory cytokine production and roles for both Th17 and Th1 in ALS progression[60]. These proinflammatory cytokine-producing CD4+ cell types were increased in ALS patients compared to controls, as well as IL-17 producing cells and total levels of IFN-y, thus suggesting that the increased proinflammatory milieu plays a substantial role in exacerbation of motor neuron death. On the other hand, T cells expressing neurotrophic factors such as BDNF were also increased, which suggested that although Th1 and Th17 Teffs dominate the response in progressive ALS, neurotrophic responses also may be involved in determining the tempo of disease progression.

In addition to the increase in Teff populations and pro-inflammatory cytokine levels associated with ALS progression, Treg (CD4+CD25+) levels are decreased in peripheral blood and are correlated with increased disease progression[61]. Since Tregs possess the capacity to harness overactive immune responses, reduction of Treg levels may indicate a deficit in the ability of the patient to suppress an overactive and aberrant immune response. To support that notion, one study found that the numbers of Tregs and FOXP3 protein expression were reduced in progressive ALS patients, and this reduction correlated with disease progression[62]. Thus, these data suggest the importance of FOXP3 to bestow a functional ability of Tregs to suppress Teff subsets, and lack of FOXP3 expression could result in aberrant immune responses that may accelerate disease progression.

Studies using animal models of ALS show that Teffs and Tregs play differing roles in the pathobiology of ALS. In a study carried out by Beers *et al*., T lymphocytes were observed infiltrating into lumbar regions of the spinal cord, as well as the cervical regions during disease progression. Initially, the T cell subset was considered Th2 due to the predominant expression of GATA3 transcription factor[63]. However, as the disease progressed, IFN-γ and T-bet were upregulated, which are indicative of a Th1 phenotype, suggesting that T cell subsets modulate with disease progression or *vice versa*. This shift may also correlate with a shift in microglial state. At an early disease stage, microglia show a protective M2 phenotype, but with disease progression, microglia shift towards a pro-inflammatory or neurotoxic M1 phenotype[64]. Also, in mutant SOD1 G93A mice, numbers of CD4+ and CD8+ T cells increase, as well as activation of microglia, and inclusion of the mutant SOD1 gene onto mice without functional T cells or CD4+ T cells accelerated disease progression suggesting T cells or CD4+ T cells are beneficial in SOD1 mice[65, 66]. However, increases in CD8+ T cells are typically seen only in late stage ALS[66], whereas CD4+ increases are seen earlier, suggesting an interplay between immune activation and neurodegeneration in this disease. In one study, knocking out CD4+ cells decreased microglial reactivity suggesting a direct interaction between CD4+ T cells and glial cell activation[66]. However, another study showed conflicting results, wherein mutant SOD1 mice lacking functional CD4+ T cells presented accelerated motor neuron degradation, suggesting the importance of CD4+ T cells for neuroprotective effects in ALS[67]. Alternatively, this data may support a role for the neuroprotective capabilities of CD4+CD25+ Tregs and potentially other CD4+ subtypes. Therapeutic vaccination for motor neuron disease has also been addressed using Copolymer 1 (Cop-1, glatiramer acetate, GA), which has been shown to mediate a protective T cell response. Treatment with Cop-1 protected motor neurons against degeneration and doubled the number of surviving neurons when compared to untreated controls[68]. On the contrary, a conflicting study showed that therapeutic vaccination with a high molecular weight derivate of GA does not alter survival and does not confer neuroprotection in mutant SOD mouse models[69].

Besides the detection of a Th1 phenotype and increased microglial reactivity in ALS mouse models, the benefits of Treg-mediated protection of motor neurons have been reported. In mSOD1 mice, Treg numbers that produce IL-4, IL-10, and TGF-β are increased in early disease onset suggesting that immunosuppressive capability may delay disease progression[70, 71]. Co-cultured with Teffs, Tregs from animals in early stages were effective at inhibiting proliferation using cytokine mediators. However, later in disease, Treg numbers were decreased, and the ability to inhibit Teff proliferation was diminished suggesting a functional deficit in those Tregs[70, 71]. Indeed, disease progression accelerated with diminished Treg function. Together, these data support the importance for Treg-mediated suppression of detrimental immune responses associated with the disease and in slowing the tempo of disease progression. Another study from our own laboratory, using T cell adoptive transfer into the mutant SOD1 G93A model, found that transfer of either activated Teffs or Tregs from wild-type mice delayed disease onset, loss of motor function, and extended survival[14]. Moreover, only transfer of Tregs delayed onset of clinical signs, whereas transfer of Teffs increased the latency between disease onset and entry into late stage disease. These results indicate that CD4+ T cells, regardless of phenotype, may induce some protection in the mouse model of ALS depending on the stage of the disease. For instance, during early stages of disease, CD4+ Tregs may enhance M2, instead of M1 microglial phenotypes. However, at end stage of disease when cytotoxic CD8+ T cell numbers are found to increase and Treg-mediated neuroprotective capabilities are diminished[67], transfer of anti-CD3 activated Teffs may function at several levels such as increased production of anti-inflammatory mediators or increased FAS-mediated lytic capabilities that can both act on either activated microglia or neurotoxic Teffs.

Another neuroprotective strategy is the selective knock down of mutant SOD1 in neuronal and non-neuronal cell types of the CNS. In order to determine whether diminution of mutant SOD levels affected disease progression, mice carrying deletable mutant genes were utilized. Deletion of mutant SOD1 from motor neurons delayed onset of disease and progression through early stage[72, 73]. Because glial activation is an accepted hallmark of mutated SOD1 in ALS, researchers began to address levels of mutant protein expression in glial cells and their effect on disease. Selective deletion of mutant SOD1 in microglia resulted in extended survival due to the significant delay of post-onset disease progression beyond that observed in selective deletion from motor neurons[72, 73]. Deletion of mutated SOD1 in astrocytes while delaying activation of microglia, did not affect disease onset or early stage disease, but delayed late stage disease progression[74]. Together, these data suggest that limiting mutant levels in both neurons and non-neuronal cell types slow disease onset and progression and increase survival. Thus therapeutic targeting of mutant SOD1 expression by microglia or astrocytes may prove beneficial in the treatment of ALS.

### Alzheimer’s disease

AD is the most common form of dementia-producing neurodegenerative disorders. Pathologically, cortical and subcortical neurons and synapses are preferentially and progressively lost with histological hallmarks of neurofibrillary tangles and extracellular amyloid-beta (Aβ) plaques[75]. The formation of these histological hallmarks is thought to contribute to neuronal death in areas such as the hippocampus and cortex resulting in several behavioral and cognitive impairments. Disease risk factors include both genetics and environment. Moreover, increased neuroinflammation due to microglial responses from neuronal loss and aberrantly cleaved and folded protein components is implicated as well as BBB dysfunction and lymphocyte infiltration[76].

Indeed, multiple studies suggest that neuroinflammation in the CNS of AD patients is associated with increased T cell infiltration[76–79]. Immune profiling of peripheral blood from AD patients shows significant aberrations in immune populations which may be associated with disease progression[80]. T cells and B cells are diminished, with substantial changes in CD4+ and CD8+ T cell populations. Larbi *et al.* have found a significant decrease in the frequencies of naïve CD4+ T cells, with concomitant increases in effector memory T cells in AD patients[81]. Frequencies of CD4+CD25+ Tregs were also reduced. Another study confirmed the decrease in total CD3+ T cells as well as decreased numbers of B cells[82]. Interestingly, CD4+ T cell subsets were increased while CD8+ T cell numbers were decreased. However, other studies have detected no significant changes in CD8+ T cell numbers or cytokine levels in AD patients[83]. Additionally, the levels of different T cell subsets detected are greatly variable, but each study documents some systemic immune aberrations in a CD4+ T cell population. One study noted, in combination with T cell subset changes, CD8+CD28- suppressor cells were decreased among PBMCs from AD patients, as well as IL-10 production[84]. These data suggest that the immunosuppressive capabilities in AD patients are diminished and could represent a deficit in the ability to control Teff responses. Similarly, increased activities of Th17 and Th9 subsets have been found in AD patients[85]. Indeed, levels of the proinflammatory cytokines IL-21, IL-6, and IL-23, and the Th17-associated transcription factor RORγ, were increased among lymphocytes in AD patients. Moreover, IL-9 was produced in significantly higher levels by cells from AD patients. Together, these data are indicative of increased levels of functional Th17 and Th9 phenotypes, which may lead to profound skewing of an inflammatory immune response in those patients.

Animal models of AD have shown increased numbers of infiltrating neutrophils, macrophages, and T cells into the CNS from the periphery, possibly through BBB dysfunction[86, 87]. Specifically for T cells, Th1 cells secreting IFN-γ and Th17 cells producing IL-17 were present in the CNS in amyloid precursor protein/presenilin 1(APP/PS1) mutant mice[88]. Interestingly, peripheral immune activation using respiratory infection also increased T cell infiltration into the brains of AD mice[89]. In those animals compared to controls, frequencies of CD3+ T cells were significantly increased as well as those of CD4+ and CD8+ T cells, proinflammatory Th1 and Th17 Teffs, microglia, and expression of pro-inflammatory genes encoding TNF-α, IL-1β, and IL-6. These increased levels of proinflammatory immune cells and mediators corresponding to increased levels of soluble and insoluble Aβ as well as increased numbers of plaques. Another study, in a rat model of AD, showed increased IL-17 and IL-22 cytokine production in the hippocampus that further supports the role of Th17-mediated promotion of microglial activation leading to increased neuroinflammation and neurodegeneration in AD and AD models[87]. Together, these data suggest that specific Teffs may provide neurotoxic conditions such that they contribute to the inflammatory cascade associated with AD and exacerbation of disease progression. Additionally, blood vessels near Aβ deposits express high levels of intercellular adhesion molecule-1 (ICAM-1) and vascular cell adhesion molecule 1 (VCAM-1), contributing to the extravasation of activated, antigen-specific (Aβ) T cells from the periphery[90]. Possibly, drainage of inflammatory mediators with aggregated and modified Aβ from the CNS provide sources of activating agents and modified self-antigen for APCs such as dendritic cells to induce Aβ-specific T cells. In turn, activated T cells could enter the CNS to inflamed sites via gradients of chemokines and cytokines that recruit immune cells or, alternatively, a few activated T cells could enter the inflamed area and in a paracrine manner, create the pro-inflammatory milieu themselves necessary to recruit and initiate other activated CD4+ T cells. On the other hand, T cell entry into areas of Aβ deposits has been observed to be beneficial in some cases[91]. Primary cell line data indicated that Th2 cells inhibit Th1- and Th17-mediated toxicities by decreasing IL-1β and IL-6 production, suggesting that this subset has regulatory and anti-inflammatory capacities. Active immunization using Aβ42 in a mouse model of AD enhanced clearance of Aβ plaques[92], and was thought to induce anti-inflammatory Th2 Teffs which increased neutralizing and clearing anti-Aβ antibodies. Therefore, to enhance Aβ clearance and downregulate the detrimental proinflammatory cascade in AD patients, an Aβ vaccine approach was thought a promising therapeutic strategy. However, after a successful phase 1 trial that immunized AD patients with Aβ1-42 peptide (AN1792) and QS21 adjuvant[93], a phase 2a clinical trial yielded a proportion of patients that experienced subacute meningoencephalitis[94]. Post-mortem evaluation of patients revealed that the vaccine had markedly cleared Aβ plaques, however neurofibrillary tangles remained[95]. Moreover, T cell infiltration and inflammation near blood vessels were also observed in post-mortem tissues, suggesting that vaccination may generate T cells capable of infiltrating the CNS and exacerbating the pathology associated with AD.

### Parkinson’s disease

PD is characterized by the progressive loss of dopaminergic neurons that originate within the substantia nigra (SN) and innervate the striatum resulting in the loss of dopamine, thus causing a majority of the motor symptoms associated with PD[96]. Lewy bodies (LB) and Lewy neurites (LN), two hallmarks of PD, are intracellular inclusions consisting of modified and misfolded alpha-synuclein (α-syn) as well as ubiquitin[97, 98]. These hallmarks present themselves in both sporadic and familial cases of PD. Familial PD accounts for approximately 10% of all PD cases and several genes have been identified in patients with a family history of PD. Six genes have been clearly linked to PD including *SNCA, LRRK2, PINK1, DJ-1, ATP13A2,* and *Parkin*[99]. While the loss of neurons and dopamine explains the motor function disturbances, the underlying driving force behind the progression of PD is still unknown. However, works performed by many researchers provide strong evidence for the involvement of the immune system in PD and neurodegeneration.

Initial studies of peripheral lymphocyte populations from PD patients showed decreased frequencies and total numbers of CD4+ T lymphocytes compared to controls[100–103]. Due to conflicting reports from only a few studies on T cell phenotypes, a firm consensus of other T cell subset changes in PD patients has proven difficult. For instance, the diminution of CD4+ T cell numbers in PD patients was found chiefly from decreased numbers of CD4+CD45RA+ naïve T cells and to a lesser extent from CD4+CD29+ memory subsets[100], whereas, Stevens and colleagues reported decreased levels of CD4+CD45R0+ memory T cells[103]. A recent study by Saunders and colleagues showed slight, yet significant increases in frequencies of CD4+CD45R0+ memory/effector T cells with concomitant diminution of CD4+CD45RA+ resting/naïve T cell levels[102]. Additionally, frequencies of peripheral CD4+ T cells with effector-associated phenotypes such as FAS + were increased in patients, whereas those expressing α4β7 integrins and CD31 (PECAM1) were diminished. Notably, these changes in CD4+ T cell phenotypes were correlated with severity of motor function as scored by the Unified Parkinson’s Disease Rating Scale, part III (UPDRS III). Differences in these immunological profiles among the few reports may range from the heterogeneity of disease to individual laboratory methodologies, but clearly require further investigation to attain consensus profiles.

Post-mortem studies of PD patient brain tissues showed both CD4+ and CD8+ T cells in close proximity to dopaminergic neurons within the SN at levels exceeding 10-fold those found in brains of controls[104]. Moreover, these levels of T cells were not detected in non-lesioned brain regions. Microarray analysis of peripheral blood leukocytes and SN brain tissue showed many genes expressed were in common with those expressed by Th17-mediated immune reactions and suggested to the authors that idiopathic parkinsonism is a Th17 dominant autoimmune disease[105]. However, whether T cell infiltration is primary or secondary to PD progression is still unclear. Similarly, conflicting reports of Tregs in PD are also wrought with variances in levels detected ranging from increased frequencies in PD patients compared to controls to little or no differences[13, 100–102, 106]. However, one study demonstrated the diminished capacity of Tregs from PD patients compared to those of controls to inhibit the proliferation of responder T cells from healthy donors[102]. This suggested that a dysfunction in Tregs leads to a hyper-activated immune state and increased disease progression. The notion that hyper-activated immune responses support increased dopaminergic loss was provided by animal studies.

Multiple studies in animal models have demonstrated the involvement of the adaptive immune system in dopaminergic neurodegeneration using both active and passive transfer of immunity[13, 104, 107]. In the 1-methyl-4-phenyl-1, 2, 3, 6-tetrahydropyridine (MPTP) mouse model, numbers of T cells in the SN are increased after intoxication, and interestingly, numbers of CD8+ T cells predominate those of CD4+ T cells. While in agreement of the relative proportions of T cell subsets within the SN of MPTP-treated mice, the total numbers of CD4+ T cells vary widely between studies. The importance of T cells to MPTP-induced neurodegeneration was found initially in adoptive transfer and reconstitution studies of functional T cells to immune deficient mice[104, 108]. While both studies confirmed that immune deficient mice were not susceptible to MPTP intoxication, reconstitution of those mice with functional naïve lymphocytes partly restored MPTP susceptibility[108] and CD4+ T cells were chiefly responsible for MPTP susceptibility[104]. These studies point to a deleterious role of CD4+ T cells in PD, unlike the beneficial role they play as described in ALS; however in the former, those T cells were determined to be Th1 and Th17 effector T cells. Of primary importance to these studies was the finding that immune T cells could augment MPTP-induced neuroinflammation and neurodegeneration verified through the use of mice lacking CD4+ T cells[104]. T cells from mice immunized with a modified self-antigen, nitrated α-synuclein (N-α-syn) recognized only N-α-syn, but not unmodified α-syn in *in vitro* challenge assays. Moreover, N-α-syn specific Teffs exacerbated neuroinflammation and increased neuronal injury and subsequent neurodegeneration of dopaminergic neurons within the SN of MPTP mice[108]. These findings indicate that N-α-syn, as a modified self-protein, either evades or breaks immunological tolerance to self α-syn and induces N-α-syn specific T cells. Indeed, by polarizing N-α-syn specific CD4+ T cells into different Teff types and adoptively transferring each separate Teff type into MPTP mice, another study showed that Th17 Teffs possess a significantly greater capacity to exacerbate dopaminergic neurodegeneration than the same number of Th1 Teffs[13]. Together, these data indicate that CD4+ T cells play an important role in the neuroinflammation and subsequent neurodegeneration in models of PD and that Th17 Teffs are more potent at direct killing of neurons or alternatively, enhancing neurotoxic microglia. These data also support the notion that peripherally circulating Teffs, as found increased in PD patients, are capable of migrating to the sites of neuroinflammation and can exacerbate and accelerate PD disease progression.

Activated microglia and Teffs are thought to be mediators of neuroinflammatory processes in PD progression. Left uncontrolled, these mediators support an inflammatory cascade that affects the tempo of disease[12]. Initially, studies directed to harness the inflammatory cascade demonstrated that adoptive transfer of CD4+ T cells from copolymer-1 (Cop-1) immunized donor mice protected dopaminergic nigral neurons and striatal termini in MPTP-treated mice[109]. These observations supported the hypothesis that subpopulations of T cells mitigate neurodegeneration in the MPTP animal model of PD. These findings are congruent with known mechanisms by which Cop-1 regulates proliferative and inflammatory responses by preferentially inducing Th2, Th3, and Tregs that secrete anti-inflammatory cytokines[12, 50, 51, 109, 110]. In a separate line of study, researchers found that CD4+CD25+ Tregs were most capable of suppressing neuroinflammation and neurodegeneration in the MPTP model with as few as 3.5 × 10^6^ Tregs being sufficient to provide virtually complete neuroprotection to dopaminergic neurons along the nigrostriatal axis[12]. Moreover, the degree of protection afforded by Tregs seems to increase with increasing inflammatory responses as evidenced by increased neuroprotection with Treg co-transfer with N-α-syn Th17 in the MPTP model[13]. Moreover, use of vasoactive intestinal peptide (VIP), a known inducer of Treg activity[99] increased the neuroprotective capability of Tregs from VIP-treated donors in the MPTP model[13]. In that same vein, studies using granulocyte macrophage colony stimulating factor (GM-CSF) showed that treating animals with GM-CSF prior to MPTP-intoxication increased Treg activity in a dose dependent fashion as well as diminished the neuroinflammatory response and provided significant dopaminergic neuroprotection[111]. Taken together, the results here and from PD patients formed the basis for a clinical strategy (ClinicalTrials.gov: NCT01882010) to target dysregulated Treg function in PD patients with GM-CSF (Leukine, sargramostim) to upregulate Treg numbers or function that will suppress neurotoxic Teff and microglial immune responses and afford a neuroprotective outcome that inhibits or slows PD progression[2, 111].

As previously discussed, each neurodegenerative disease has different pathological hallmarks. The cellular location of these hallmarks varies from primarily intracellular in PD and ALS to primarily extracellular debris in MS and AD; however it should be noted that intracellular inclusions eventually become extracellular, especially upon neurodegeneration. Additionally, T cells generally do not recognize and respond to extracellular antigen and debris, but depend on the presentation of an antigen in the context of MHC or to bystander effects from glial cells. To our knowledge, no studies implicate different types of T cells or T cell responses in relation to both intracellular and extracellular pathological hallmarks. Thus, an interesting hypothesis asserts that the extent of external debris processed during disease progression could influence the overall scope of T cell responses. A most notable ramification of this notion is the putative importance that animal models do not present all the pathological hallmarks distinct for each disease. Nonetheless, animal models do provide researchers with the ability to assess pathology, etiology, and overall disease progression within the limits of the model, and represent indispensable tools to develop and test potential therapeutics.

## T cell migration to sites of inflammation

The adaptive immune system functions to aid in effective clearance of foreign antigens by recognizing antigen presented in the context of an MHC molecule. Most efficacious immune responses occur in close proximity to the foreign antigen (*e.g.*, cell-mediated lysis of infected cells). To gain proximity, circulating lymphocytes migrate across luminal barriers to gain entry to sites of infection or inflammation. Successful extravasation of an activated T cell from circulation to an area of inflammation is dependent on cell adhesion molecules (CAMs) and CAM ligands. These specialized binding partners expressed on both circulating T cells and endothelial cells (ECs) are critical to recruitment of proper immune cells to the site of inflammation. However, due to the BBB, migration of cells into the CNS and inflammatory sites therein presents a formidable exercise in mobility. The EAE model provides a prototypical system for the successful interaction of antigen specific CD4+ effector T cells with ECs and altered CAM expression to facilitate entry into the CNS and cerebral spinal fluid (CSF)[6, 112, 113].

### Disease and CAM expression

T cell migration across any EC barrier, and particularly across the BBB requires a distinct set of steps; each step mediated by different CAMs on both T cells and ECs[6, 114]. Under normal conditions, expression of CAMs by brain ECs is minimal or completely lacking[6–8, 115]. However, under inflammatory conditions, ECs are activated by cytokines and upregulate CAM expression. Assessing the infiltration of circulating lymphocytes across ECs has shown that the expression of CAMs and cell adhesion ligands changes following antigen activation and/or cytokine release. These changes typically precede disease onset and either increase or decrease binding of circulating lymphocytes to target endothelium. Furthermore, studies have shown that, under disease states including MS, viral encephalitis, and stroke, specific CAMs are upregulated[7, 8] lending evidence that the immunological processes associated with these diseases could be mediated through increased expression of CAMs, and thus, provide strategic targets for therapy. Indeed, the ability to transfer EAE using myelin basic protein (MBP)-TCR transgenic T cells was dependent on the expression of glycosylated ligand for P-selectin, PSGL-1[9, 116]*.* This ability was proposed to correlate with the upregulation of PSGL-1 on their surface. In MS patients, greater numbers of peripheral CD4+ T cells are found that express higher surface levels of PSGL-1 than in controls, suggesting an enhanced capacity to interact with CNS ECs in a PSGL-1 dependent manner[6, 9]. Another adhesion molecule previously described as a novel marker associated with tumor metastasis, melanoma cell adhesion molecule (MCAM) was found to play a role in migration of Th17 cells in EAE[117].

### Antigen-induced CAMs

The co-culture of lipopolysaccharide (LPS), a component of gram-negative bacteria, with ECs induces upregulation of multiple CAMs on the luminal side (ICAM-1, VCAM-1, endothelial (E)-selectin) in a time and concentration-dependent manner[118, 119]. These data, combined with the sensing of LPS as MAMPs, show that induction of microglial activation, neurotoxic factor production, and significant dopaminergic neurodegeneration provided evidence for LPS as an animal model of nigral dopaminergic neurodegeneration[113, 120–122]. Other molecules also have shown similar capabilities as LPS. Within the SN of PD patients, N-α-syn accumulates within neurons and is one component of Lewy bodies. When released into the extraneuronal environment, microglia detect N-α-syn as DAMPs and elicit a proinflammatory phenotype that is neurotoxic and accelerates the death of dopaminergic neurons[59, 123–126]. The ability to induce a proinflammatory response by microglia suggests that aggregated or N-α-syn may act as a neo-antigen and stimulate APC function by dendritic cells in draining tissues or even resident brain microglia resulting in sustained activation of microglia[108, 127]. Moreover, those studies showed within 20 hours after MPTP-intoxication, N-α-syn is detected within draining cervical lymph nodes, wherein APCs are upregulated and later elicit peripheral adaptive immune responses. Furthermore, evidence that N-α-syn-specific CD4+ Teffs accelerate disease progression was suggested by increased neuroinflammation and subsequent dopaminergic neurodegeneration after adoptive transfer of N-α-syn specific T cells. However, the mechanism(s) by which those Teffs function and whether they must cross the BBB are not entirely understood. Models that address a prominent role of CD4+ Teffs in driving inflammation implicate infiltrating Th17 Teffs that possess unique migratory properties and expression of MCAM for tissue entry[117]*.*

### Aβ as an inducer of CAM expression

While the exact mechanism of T cell infiltration across the BBB remains unknown in AD, multiple studies have addressed this topic. As discussed, patients with AD display large accumulations of Aβ plaques in the brain, and Aβ-specific T cells have been shown to effectively migrate across the BBB. In an APP/IFN-γ Tg mouse model, Aβ immunization resulted in migration of both CD4^+^ and CD8^+^ T cells into the brain parenchyma[128]. Quantitation and analysis showed the majority of those T cells were antigen-specific, CD4^+^ T cells with a Th1 phenotype, characterized by their predominate secretion of IFN-γ. Thus far, the mechanism by which circulating lymphocytes migrate into the CNS in AD is unclear, but upregulation of different CAMs associated with transendothelial migration has been documented in both animal models of AD and AD patients. For instance, peripheral T cells from AD patients overexpress macrophage inflammatory protein-1α (MIP-1α), which induces expression of CCR5 on human brain microvascular endothelial cells, ultimately leading to enhanced migration across an artificial BBB[129]. Another study showed that compared to age-matched controls expression of CXCR2 was increased on peripheral T cells from AD patients[130]. In that study using AD models, increased expression of CXCR2 was dependent on levels of microglial-derived TNF-α and enhanced T cell entry into the CNS. In both studies, inhibition of the receptor-ligand interactions through the use of antibodies or antagonists effectively blocked T cell entry in transendothelial migration assays, further supporting the concept that blockade of cellular adhesion molecules in AD could inhibit extravasation of pro-inflammatory, and possibly detrimental, antigen-specific T cells into the CNS. In a later study, the role of enhanced CCR5 expression in promoting T cell migration was verified in migration assays, and further showed that Aβ interaction with RAGE, an Aβ receptor, induces CCR5 upregulation on brain endothelial cells[79, 129]. Moreover, animal studies showed that intracerebroventricular injection of CD4+ T cells into the brain parenchyma increases ICAM-1 expression by brain endothelial cells, further suggesting that a putative mechanism for T cell transmigration in AD would encompass CAM modulation and interaction by both T cells and endothelial cells[131].

### Mutant α-synuclein and CAMs

Two studies demonstrated that wild type (WT) α-syn and mutant forms of α-syn (A30P; E46K; A53T) can alter the expression levels of ICAM-1 and CD44 on human astrocytes, astrocytic cell lines, and murine microglial cell lines[10, 11]. The upregulation of CD44, a binding partner for E- and P-selectin was shown to correspond with an increased migratory capacity of microglial cells both *in vivo* and *in vitro*. Upregulation of CAMs by WT and mutant α-syn can regulate microglial migration into the SN, accelerating the pathogenesis of PD[10, 11]. These studies provide evidence that α-syn is capable of altering CAM expression and that a therapeutic approach to block CAM expression could provide a beneficial outcome in PD patients.

### CAM involvement in T cell infiltration to CNS

Activated CD4+ T cells, compared to naïve CD4+ T cells, circulate within the vasculature differently[132] and have the capacity to cross the BBB. Studies using adoptive transfer of MBP-specific T cells in the EAE model showed that VCAM and ICAM-1 were upregulated by those cells by 48 hours after transfer[98]. In MS lesions, altered levels of adhesion molecules and their respective ligands have been identified on endothelial cells and perivascular inflammatory cells[27]. Specific blocking of either α4-integrins or VCAM-1 inhibits the development of EAE, indicating that α4-integrin interactions with VCAM-1 play a critical role in the recruitment of Teffs across the BBB[133, 134]. Natalizumab, a drug indicated for relapsing-remitting MS, is a human monoclonal antibody that specifically blocks the α4 subunit of integrins and has yielded favorable clinical outcomes[135]. A marked increase in ICAM-1 was found within the SN at sites of T cell infiltration following MPTP-intoxication[104] and numbers of peripheral CD4+ T cells that expressed α4β7 integrin were diminished and inversely correlated to clinical severity in PD patients[102] suggesting that Teffs are removed from the peripheral circulation, either by elimination or dissemination to sites of CNS inflammation, and may play a role in disease progression. However, until recently, no studies have adequately addressed or evaluated CAM changes within the SN of PD patients or in mouse models of PD.

## Mechanisms of T cell-mediated destruction and protection

**Figure 1:**
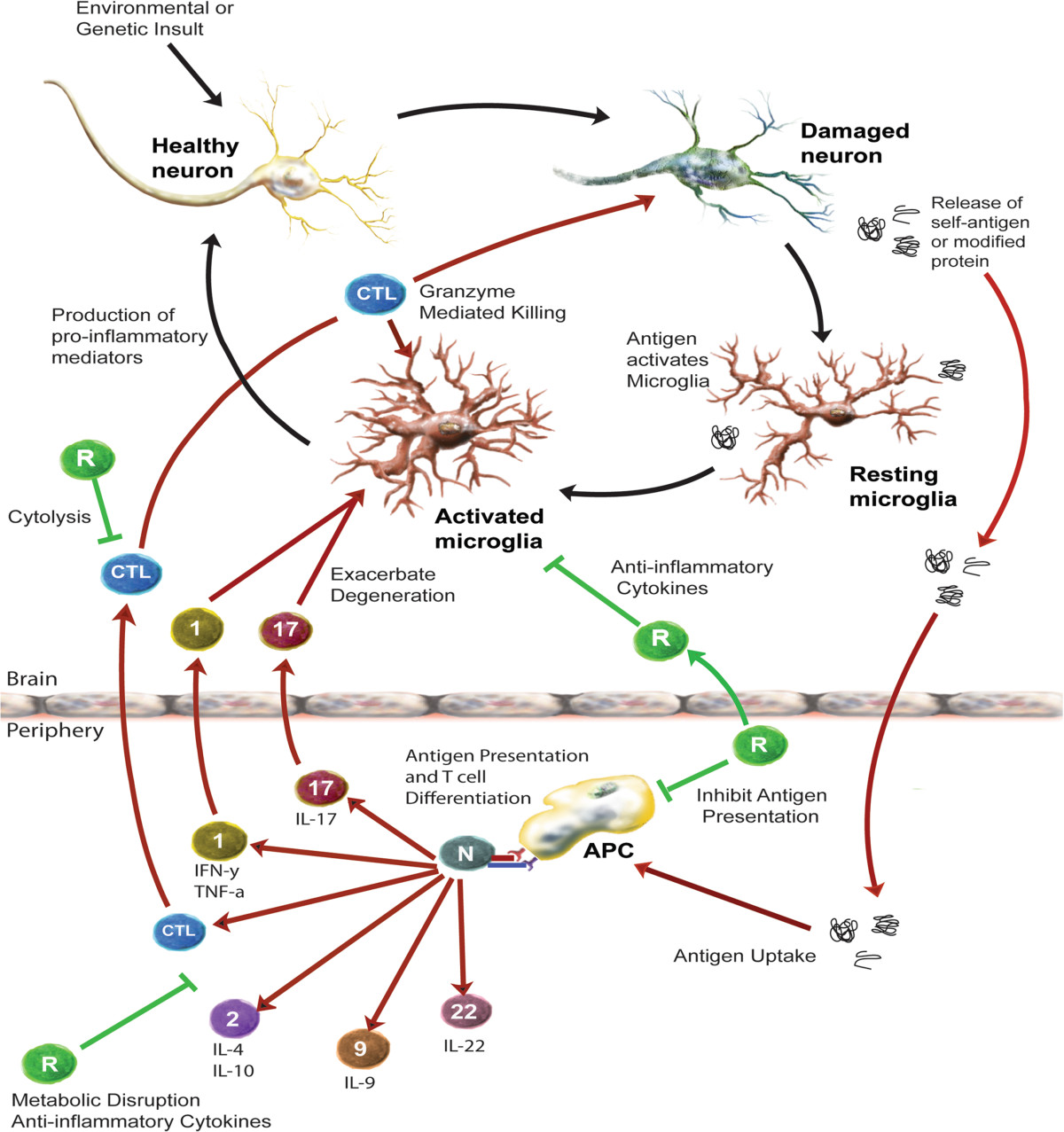
**The immune system and neurodegeneration.** Death or damage of neurons can be mediated via several mechanisms in the CNS. Upon insult, healthy neurons become damaged, causing release of self-antigens or modified proteins. These antigens remain in the CNS to activate surrounding resting microglia to an activated phenotype. Reactive microglia produce proinflammatory mediators such as neurotoxic cytokines and reactive oxygen and nitrogen species (ROS/RNS), increase oxidative stress, and further contribute to neuronal damage. Modified and misfolded self-proteins that drain into secondary lymphoid tissues are phagocytized, processed, and presented on MHC by APCs to naïve T cells (N). Upon recognition of antigen, T cells differentiate into antigen-specific T effector (Teff) or T regulatory (Treg) phenotypes. Teff subsets include Th1 (1), Th2 (2), Th9 (9), Th17 (17), Th22 (22), and cytotoxic T lymphocytes (CTLs). Additionally, reactive microglia signal proximal endothelial cells by cytokine and chemokine gradients to upregulate CAMs. In turn, activated Teffs such as Th1 and Th17 with upregulated integrins and CAM ligands bind CAMs via CAM-ligand interactions and extravasate across the BBB. Upon recognition of modified self-antigen presented by MHC of microglia/macrophages, activated Teffs generate neurotoxic and proinflammatory factors that drive M1 microglia or resting microglia to a higher reactive state and support a neurotoxic cascade. CD4+ Th1 or Th17 Teffs induce FAS ligand or produce neurotoxic cytokines such as TNF-α, IL-17, and IFN-γ that may directly interact with cognate receptors expressed by neurons. CD8+ CTLs can recognize antigen/MHC I complexes on neurons to induce perforin- and/or granzyme-mediated cytolysis. In response to inflammatory events, Tregs (R) attempt to counteract the neurotoxic cascade through inhibition of antigen presentation, production of anti-inflammatory cytokines, metabolic disruption, cytolysis of Teffs or reactive microglia, and induction of neurotrophic factors by astrocytes; all mechanisms aim to interdict the neuroinflammatory-neurodegenerative cycle and ultimately support neuronal survival.

Based on interactions between T cells and surrounding immune cells, T cells can play a neuroprotective or neurodegenerative role in several diseases discussed (Figure 1). The cross-talk between T cells and glial cells is thought to help mediate effector functions by either cell-cell contact or cytokine-mediated mechanisms. Possible neurodestructive mechanisms include direct cytotoxicity by proinflammatory cytokines, hyperactivation of proinflammatory microglia, or diminished suppressive function of Tregs. On the other end of the spectrum, targeting T cells to elicit a protective mechanism, could diminish the extent of neuroinflammation and therefore increase the number of surviving neurons in the CNS of patients with neurodegenerative disorders. The number of potential targets to elicit neuroprotection is extensive and will be discussed in further detail in the following sections.

### T cell-glial interactions and neurodegeneration

During viral infections and neuroinflammation, MHC II is upregulated by microglia, whereas MHC I is constitutively expressed by most cells including oligodendrocytes, neurons, microglia, and endothelia[136]. Therefore, neurons in a pro-inflammatory environment could serve as targets for CD8+ T cells with direct killing of neurons through antigen-specific interactions mediated by cytotoxic granzyme release[136]. In animal models of MS, studies demonstrated that CD8+ CTLs form stable adhesions with neuritis in a MHC I peptide-dependent fashion. Furthermore, MBP-specific CD8+ T cells induce direct tissue damage when injected into irradiated recipient mice[137] (Figure 1). Increased numbers of CD8+ T cells are found in close proximity to activated microglia in post mortem studies of PD patients[104], however their role in PD and PD animal models remains enigmatic. The presence of T cells within the brain of PD patients exceeds those typically found in the CNS and suggests a role of the T cell beyond normal surveillance[138]. These studies, taken together, suggest a strong role of MHC I restricted CD8+ T cells in the pathogenesis of neurodegenerative disorders that may involve not only disease progression, but also initiation of disease events.

The probability exists that multiple T cell subsets other than CD8+ Teffs play a role in inflammation-associated neurodegenerative disorders (Figure 1). MHC II restricted CD4+ Teffs appear to play a detrimental role in neurodegenerative disease pathogenesis. CD4+ Teffs recognize antigen presented by MHC II expressing microglia within the CNS resulting in the activation of resident immune cells. Examples of the self-reactive antigens believed to be involved in T cell activation include N-α-syn in PD[13], Aβ in AD[88], and MBP in MS[139] (Figure 1). These microglial-T cell interactions induce reactive microglia to release neurotoxic factors that ultimately damage the neuron and drive the neuroinflammatory cycle. Whereas one EAE study showed CD8+ T cells in close proximity to activated microglia and that CD8+ T cells induce direct neuronal damage via recognition of antigen in a MHC dependent fashion, T cells in another study were detected directly attached to neurons, although independent of MHC molecules, and elicited cytotoxicity via a glutamate-mediated mechanism[140]. This suggests a possible direct neurotoxic T cell-mediated mechanism that may not require recognition of self-antigen in the context of MHC. The association of T cell-mediated enhanced microglial function was recently demonstrated by depletion of CD4+ T cells from SOD-1 mutant mice that resulted in significant diminution of CD11b+ immunoreactivity, thus supporting the idea that direct T cell-microglia interactions may potentiate neurodegeneration[141]. As discussed previously in this review, CD4 knock out (KO) mice show increased survival and decreased motor loss which may suggest a dual role of CD4+ T cells in ALS pathogenesis[66]. The detrimental effects of Th17 cells are known in neurodegenerative diseases and their respective models[142]. An underlying mechanism of Th17-mediated neurotoxicity is through direct contact with neurons through Fas-Fas ligand (FasL) interactions resulting in apoptosis and neuronal death[87]. Glial cells within the CNS can also act through the Fas-FasL interactions to inhibit or kill infected cells and target activated lymphocytes or Teffs that could yield neurotoxic events[1]. Thus, the loss of potentially neurotoxic immune effector cells would eliminate those Teff-mediated killing mechanisms that act directly on neurons or indirectly via microglial activation and should prove to be neuroprotective. Furthermore, studies have shown that deficits in this regulatory circuit are known to contribute to inflammatory neurodegeneration[1].

Potentiating or mitigating neuroinflammatory circuits are astrocytes, a glial subset that normally is involved in the maintenance of brain homeostasis. However, in the presence of hyper-activated microglia, astrocytes can directly participate in inflammatory reactions to secrete pro-inflammatory mediators such as IL-6 and TNFα, upregulate expression of FasL, and diminish trophic activities that eventually lead to the acquisition of a neurotoxic phenotype that exacerbates neurodegeneration[143, 144]. Similarly, while microglia that exist in an alternatively-activated (M2) state are generally considered neuroprotective and release anti-inflammatory cytokines as well as neurotrophic factors, under neuroinflammatory conditions, microglia can readily switch to an M1 cytotoxic phenotype (Figure 1). The phenotypic switch is accompanied with upregulation of oxidative stress generating enzymes such as iNOS, NADPH oxidase, and myeloperoxidase; increased levels of reactive oxygen species (ROS) and reactive nitrogen species (RNS); and secretion of neurotoxic levels of proinflammatory cytokines including IL-1β, TNFα, and IFN-γ[64] that lead to secondary neuronal damage[145]. A recent study showed that in MS patients proportions of Th22 cells correlated with those of Th17 cells, serum IL-22 concentrations were highest during peak phases of disease, and those levels diminished during recovery phases[146]. In addition to an essential role for Th22 and Th17 in MS, these data suggested that the two Teff types may play synergistic roles in disease progression and yielded speculation that since IL-23 promotes IL-17 and IL-22 secretion, IL-23 receptor and STAT3 signaling may provide a key pathway in the activation of Th22 cells and Th17 Teffs. Moreover, activated microglia proved to be a potent source of IL-23 expression in active and chronic active MS lesions[147], suggesting that microglia may provide the stimulatory signals responsible for activation of Th17 and Th22 Teffs.

### Treg targeted neuroprotection

As we have discussed, T cells play a dual role in neurodegeneration and neuroprotection during CNS disorders. Early studies in models of nerve injury showed improved recovery that coincided with the presence of activated immune cells[145]. Therefore, targeting the adaptive immune system could provide a potential strategy to halt neurodegenerative progression. Tregs are potent immune modulators with the ability to suppress the immune system through multiple mechanisms including secretion of anti-inflammatory cytokines that inhibit differentiation of Teffs; direct killing of Teff subsets; blocking of co-stimulation of naïve T cells and Teffs; and metabolic disruption of Teffs via uptake of IL-2[23, 142] (Figure 1). Anti-inflammatory cytokines, such as IL-4, IL-10, and TGFβ, are prime anti-inflammatory mediators that diminish neuroinflammation and increase neuroprotection. Tregs isolated from mutant SOD-1 mice and co-cultured with wild type microglia suppressed NOX2 and iNOS production via an IL-4 mediated mechanism[70]. Moreover, adoptive transfer of those Tregs to T cell deficient ALS mice augmented neuroprotection of motor neurons and extended recipient survival. Additionally, neuroprotective mediators such as T cells that express anti-inflammatory cytokines and neurotrophic factors were expressed in a temporally and spatially distinct fashion depending on the clinical scores and extent of histopathology[63, 70]. Importantly, numbers of Tregs and FOXP3 expression from ALS patients were reduced in rapidly progressing ALS patients, inversely correlated with disease progression rates, and were predictive of future rapid progression[62]. Multiple lines of evidence from our laboratories have demonstrated the efficacy of several immunomodulators in eliciting Treg-mediated neuroprotection in models of neurodegeneration[12–14, 51, 109, 111, 148, 149]. These immunomodulatory agents such as anti-CD3, VIP and GM-CSF primarily act to increase Treg numbers and functional capabilities that exploit Treg function to diminish the neuroinflammatory cycle and provide neuroprotection.

### Modulation of T cell CNS infiltration

**Figure 2:**
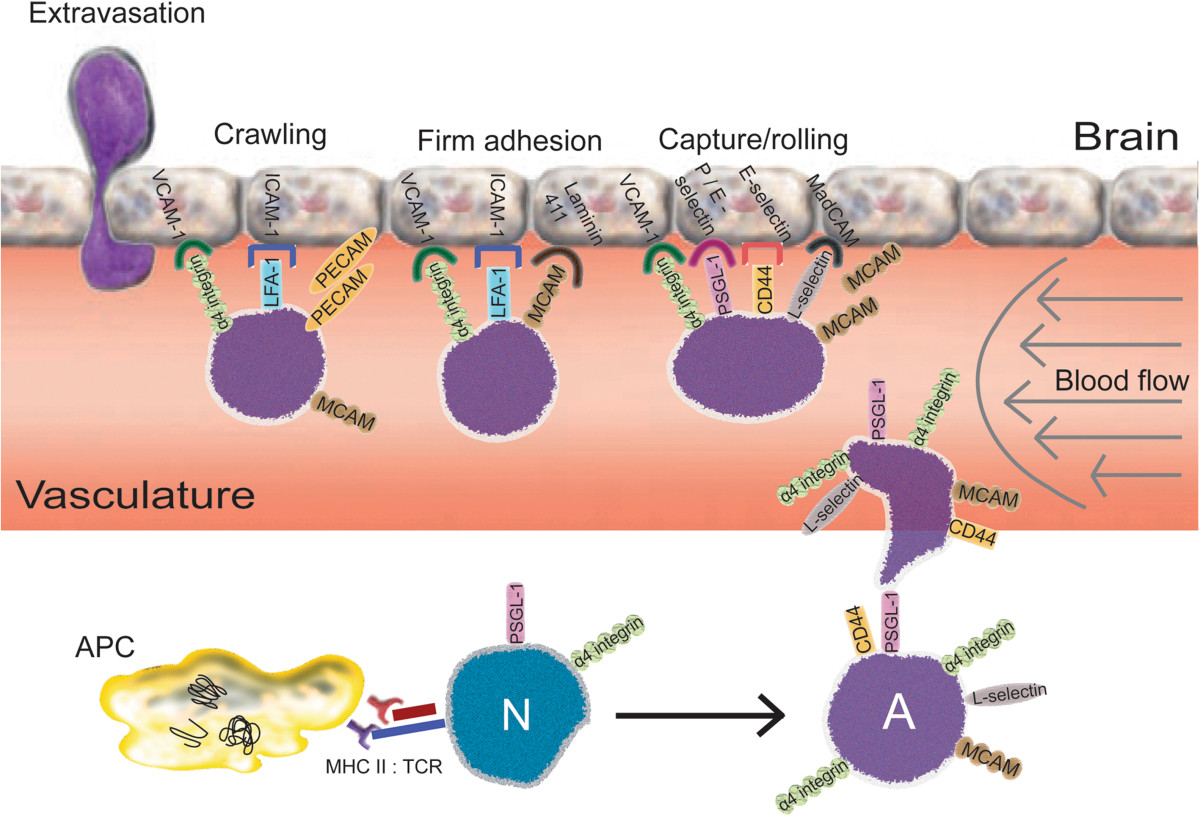
**Migration of activated T cells into brain.** Peripherally, naïve T cells (N) encounter APC that present peptides from aberrant, misfolded, or aggregated proteins associated with neuroinflammatory processes. Upon presentation of antigen and delivery of appropriate co-stimulatory signals by APC, naïve T cells recognizing the antigen/MHC complex via the TCR become activated (A) leading to upregulation of CAMs on the T cell surface. These receptors and ligands include, but are not limited to, integrins, MCAM, and PSGL-1. Similarly, at sites of neuronal injury and neuroinflammation, danger signals, pro-inflammatory cytokines, and chemokines induce upregulation of endothelial associated CAMs on the basolateral side of the blood brain barrier. Following upregulation of CAMs, activated T cells (such as pro-inflammatory, anti-inflammatory or regulatory T cells) enter the vasculature and begin the process of extravasation via either a trans- or para-cellular route. This migratory process occurs in a step-wise manner beginning with T cells loosely tethering to endothelial cells via the binding of T cell ligands to selectins, such as E-selectin and other CAMs, such as VCAM, ICAM, and laminin 411 on the luminal side of the endothelial cells. Loose tethering allows the cell to roll along the luminal side of the endothelium and interact with CAMs, pulling it closer to the endothelial cell layer to eventual capture. Upon clustering of receptors and ligands on T cell and endothelial cell surfaces, the T cell begins "crawling" across the endothelial surface until reaching an endothelial cell junction, which signals the initiation of extravasation. Transmigration proceeds, via a chemotactic gradient allowing antigen-specific T cells entrance to the brain. Once in the parenchyma, activated T cells recognize antigen presented by MHC, initiating the efferent response program of the T cells to deliver either effector or regulatory function that supports the respective neurodegenerative or neuroprotective outcome.

T cell infiltration of the CNS has been demonstrated in multiple neurodegenerative diseases[150]. This infiltration into the CNS is generally restricted to activated T cells and occurs in a well-characterized and stepwise manner[151] (Figure 2). CNS degenerative disorders wherein T cell extravasation is thought critical for disease initiation and progression is best documented in the EAE model. Blocking antibodies and multiple drugs have been utilized to target specific subunits of CAMs with the ultimate goal of blocking BBB migration of encephalitogenic Teffs that are specific for the self-antigen[27, 135, 151]. One consensus is that extravasation of T cells in inflammation-associated neurodegenerative disorders such as MS is dependent not only on the CAMs and ligands utilized by ECs and T cells, but also on the cellular architecture and the site at which T cells migrate. This consensus is particularly critical to the notion that migration of T cells across the BBB is necessary for disease initiation or progression; however, others have suggested that T cells may function in an endocrine or paracrine fashion across a BBB that has become more permeable under inflammatory conditions[152]. In addition to the conventional group of CAMs that regulate T cell extravasation, a recent study identified MCAM (CD146) expressed on Th17 Teffs and its ligand, laminin 411, expressed within the vascular endothelial basement membrane[117]. Anti-MCAM antibodies inhibited the *in vitro* interaction of MCAM and laminin 411, and administration of anti-MCAM antibodies to recipient mice prior to adoptive transfer of encephalitogenic Th17 Teffs reduced Th17 cell infiltration and ameliorated disease in EAE. These data suggested that MCAM expressed by Th17 Teffs may provide a strategic therapeutic target for inhibiting migration of neurotoxic Th17 Teffs and affording increased neuroprotection.

## Novel insights for T cell responses in Parkinson’s disease

Our laboratories have accumulated a substantial amount of evidence indicating that adoptive transfer of N-α-syn-specific Teffs after MPTP intoxication exacerbates neuroinflammation, enhances neurodegeneration, and prolongs lesion development[2, 13, 108, 111, 127]. Recent evidence from clinical studies indicated that T cells with an activated or memory/effector phenotype are present in greater frequencies in PD patients compared to age- and environment-matched caregiver controls[102]. Increased proportions of those T cell subsets were directly correlated with diminished motor function and associated with diminished Treg function in PD patients. Taken together, the detection of CD4+ and CD8+ T cells within the SN of mice treated with MPTP and in PD patients, the proximity of infiltrating T cells to MHC expressing microglia/macrophages, and CD4/CD8 ratios of infiltrating T cells that are reversed from those expected in peripheral circulation[13, 51, 97, 104, 108] provide strong evidence for the directed extravasation and migration of activated T cells to sites of inflammation and for the association of increased disease or lesion progression with increased T cell infiltration. However, whether extravasation and migration of T cells are necessary and the mechanism(s) associated with T cell migration in Parkinsonism have not been adequately evaluated.

### MPTP intoxication increases T cell migration and infiltration into the CNS

**Figure 3:**
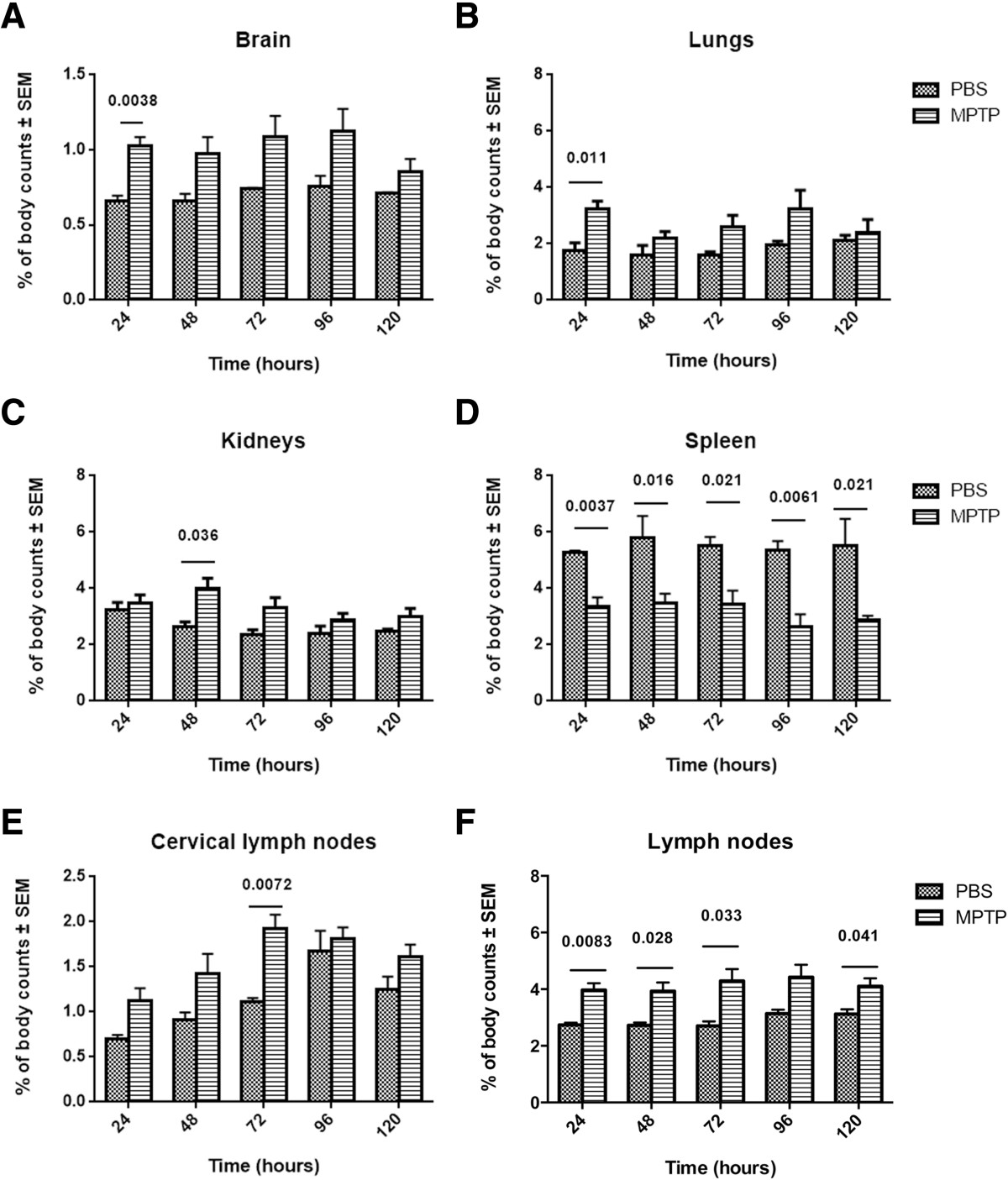
**MPTP-intoxication increases T cell migration.** CD3+ T cells were obtained and enriched from spleen and lymph nodes of male donor C57BL/6J mice. Isolated T cells were activated with anti-CD3 for 3 days. Syngeneic recipients were treated with 4 doses of MPTP-HCl in PBS (18 mg/kg, based on freebase MPTP) or PBS alone; each dose administered at 2 hour intervals. Activated T cells were labeled with ^111^In-oxyquinoline (GE Healthcare), and 20 ×10^6^^111^In-labeled T cells were adoptively transferred to each MPTP- or PBS-treated recipient. CT/SPECT images from each animal were acquired at 24, 48, 72, 96, and 120 hours post-transfer. For each mouse at each sampling time, electronic bit maps were drawn to circumscribe **(A)** brain, **(B)** lungs, **(C)** kidneys, **(D)** spleen, **(E)** cervical lymph nodes, **(F)** all other lymph nodes, and entire body. Counts of radiolabeled T cells were determined by digital image analysis software (VIVID, GE Healthcare) and corrected for decay from the time of labeling. Counts for each organ were normalized as the percentage of total body counts for each time **(A-F)**. Means ± SEMs of radiolabel percentages were determined for 3–5 mice/treatment group and differences between the 2 treatment groups were determined by Student’s t-test where p ≤0.05 was considered significant.

To examine T cell migration under neuroinflammatory and neurodegenerative conditions, mice were treated with 4 doses of MPTP, one dose every 2 hours. This dosage of MPTP induces peak neuroinflammation and rate of neuronal death by 48 hours of administration, both are typically resolved by 4 days after treatment[97, 153, 154]. Anti-CD3-stimulated and ^111^In-labeled Teffs were adoptively transferred to MPTP- or PBS-treated recipients. Animals were monitored by CT/SPECT every 24 hours for 120 hours post- transfer. We previously demonstrated that CD3 stimulated, ^111^In-labeled effector T cells did not migrate into the CNS of naïve mice (data not shown). In contrast, MPTP-treated mice consistently showed increased percentages of radiolabel in the brain compared to PBS-treated controls; however significant differences were detected only after 24 hours post-transfer (Figure 3A). Significant accumulation of labeled T cells were found in lymph nodes of MPTP mice at most times (Figure 3F), but only at 72 hours for cervical lymph nodes (Figure 3E). Differences in brain and lymph nodes, 24 hours after adoptive transfer of ^111^In-labeled Teffs, can be seen in movies of CT/SPECT-imaged recipient mice that were treated with either PBS (Additional file1) or MPTP (Additional file2). These data suggested that activated T cells can preferentially migrate to the CNS and peripheral lymphoid tissues under inflammatory conditions. In contrast, significantly greater accumulation of radiolabeled T cells were detected in spleens from PBS-treated mice than in MPTP mice (Figure 3D). While accumulation of fewer labeled T cells was found in the spleen compared to other peripheral lymphoid tissues, activated T cells could preferentially accumulate in more inflamed tissues or remain in circulation rather than accumulating in the spleen. In a previous study using only 5 × 10^6^^111^In-labeled activated Teffs and assessing CT/SPECT at 24, 48, and 72 hours, no significant differences in percentages of radiolabeled T cells could be discerned between MPTP- and PBS-treated mice in any tissue at any time (data not shown). Since adoptive transfer of nitrated α-synuclein peptide (N-4YSyn)-specific Teffs exacerbate neuroinflammation and neurodegeneration in MPTP-treated mice[13, 108], the previous data suggested the possibility that a threshold number of T cells in the brain is required for detection by CT/SPECT or that T cells remaining in the encephalic vasculature were masking those that migrated into the parenchyma.

### CAM expression increases with MPTP intoxication and adoptive transfer of T cells

**Figure 4:**
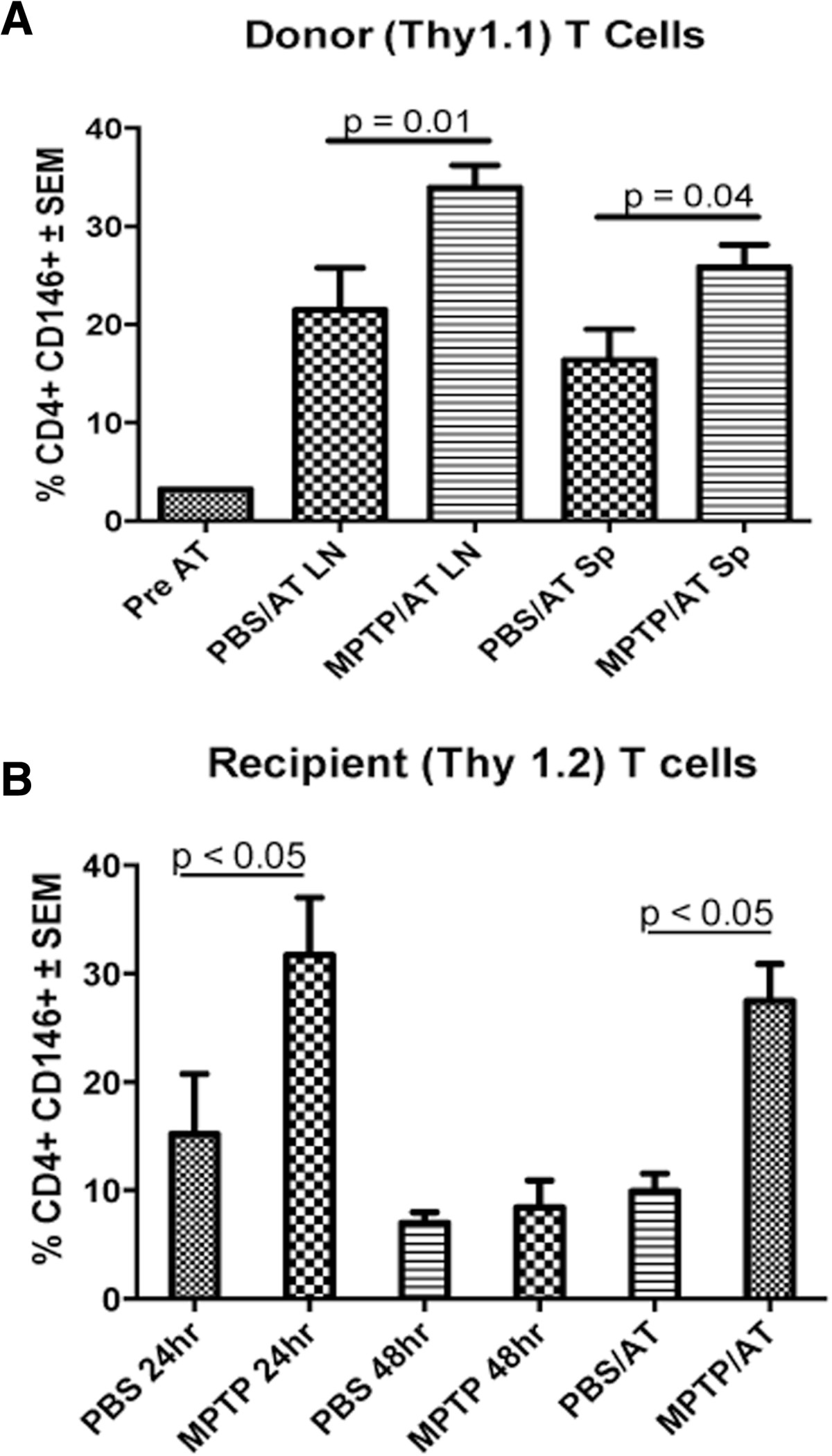
**Expression of MCAM (CD146) parallels MPTP treatment and adoptive T cell transfers.** CD3+ T cells were obtained from donor mice expressing CD90.1 (Thy1.1) and were activated with anti-CD3. **(A)** Following activation, and prior to adoptive transfer (AT), donor cells were analyzed for co-expression of CD4 and CD146 (CD4+CD146+) (Pre AT). Activated donor T cells (Thy1.1) were adoptively transferred to recipient mice expressing CD90.2 (Thy1.2) after treatment with MPTP at dosages of 18 mg/kg every 2 hours for 4 doses or with PBS. Thus, detection of Thy1.1 or Thy1.2 by flow cytometric analysis differentiates donor (adoptively transferred) and recipient (endogenous) T cells, respectively. **(A)** Forty-eight hours after adoptive transfer (AT), spleens (SP) and lymph nodes (LN) were removed from recipient animals and cells were analyzed by flow cytometric analysis for percentages of CD4+CD146+ T cells among donor (Thy1.1) T cells. **(B)** Twenty-four or forty-eight hours after PBS- or MPTP-treatment, lymph nodes were removed from mice that did not receive adoptive transfer, and cells analyzed by flow cytometric analysis for percentages of CD4+CD146+ T cells among the endogenous Thy1.2+ T cells. Additionally, 48 hours after adoptive transfer, lymph nodes from recipient mice were removed and analyzed for percentages of CD4+CD146+ T cells among the endogenous recipient Thy1.2 T cells. Means ± SEMs were determined from data within the 95% confidence intervals of the means for n =4-5 mice per group and were compared by one-way ANOVA with Fisher’s LSD post-hoc test where p ≤0.05 was considered significant.

Altered expression and recognition of CAMs and ligands on ECs and leukocytes are the primary mechanisms by which activated T cells extravasate from circulation to brain parenchyma[151]. One such CAM that is associated with expression by Th17 Teffs is MCAM (CD146), a member of the immunoglobulin superfamily of CAMs[155]. Expression of MCAM by CD4+ Th17 cells has been implicated in playing a major role in the extravasation of Th17 cells in EAE[117]. Therefore, these studies sought to evaluate MPTP-induced alterations in the expression of MCAM on activated T cells. Donor CD3+ T cells (Thy 1.1 or CD90.1) were activated with anti-CD3. Activated donor T cells (Thy1.1) were adoptively transferred to MPTP- or PBS-treated recipients (Thy 1.2 or CD90.2). Spleens and lymph nodes were removed from recipients and analyzed by flow cytometric analysis for expression of CD4+CD146+ T cells within the transferred donor (Thy1.1) or the endogenous recipient (Thy1.2) T cell populations. Prior to adoptive transfer, little expression of CD146 was detected among the stimulated donor T cells (Figure 4A). CD146 expression increased among CD4+ donor T cells by 48 hours post-transfer to either PBS- or MPTP-treated mice. Interestingly, frequencies of CD146+ T cells were increased among donor T cells from MPTP-treated recipients compared to those from PBS controls. While no differences in frequencies of CD4+CD146+ T cells were detected among endogenous (Thy1.2) splenic T cells regardless of treatment (data not shown), significant differences were found in endogenous T cells from lymph nodes (Figure 4B). By 24 hours post-MPTP or 48 hours post-adoptive transfer with MPTP-intoxication, percentages of endogenous T cells expressing CD146 significantly increased. However, by 48 hours post MPTP-intoxication in the absence of adoptive transfer, MCAM levels diminished to control levels, suggesting that MCAM expression is upregulated quickly, but transiently expressed after an initial insult (*e.g.,* MPTP intoxication) as are other CAMs[156], and that activated T cells after homing to sites of accumulation or inflammation may prolong the expression of MCAM by endogenous T cells. Taken together, these findings indicate that adoptive transfer of activated T cells to MPTP-treated recipients is sufficient to upregulate MCAM expression on activated donor T cells as well as recipient T cells; however, in the absence of initial insult, expression is diminished or transient.

### Potential role of MCAM in T cell migration

**Figure 5:**
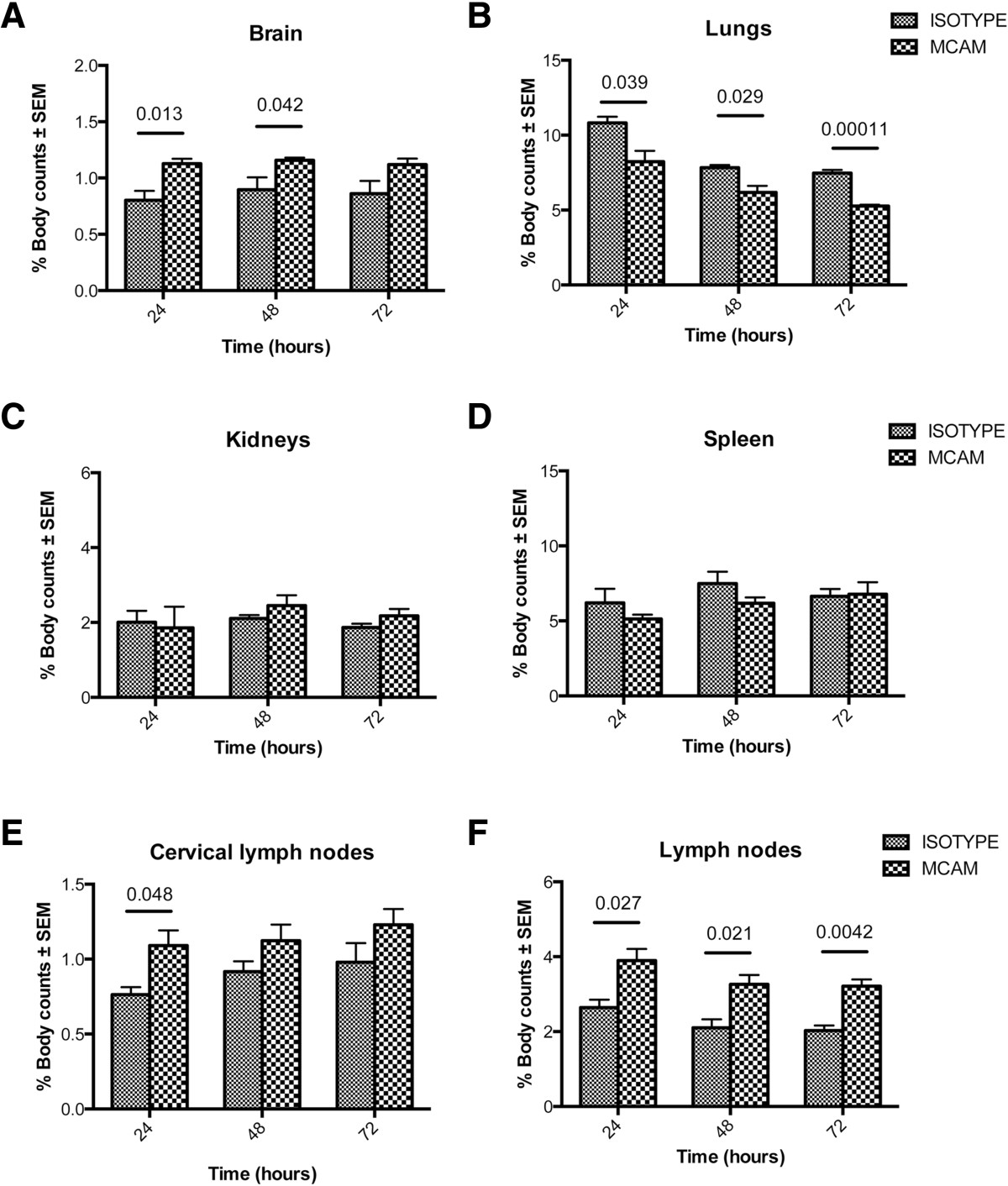
**Anti-MCAM treatment affects T cell migration to the lungs but not brain.** Donor CD4+ cells were isolated from spleens and lymph nodes of C57BL/6 male mice. T cells were activated and polarized by culture for 5 days in the presence of anti-CD3 and a Th17-polarizing cocktail (3 ng/ml TGF-β, 10 ng/ml IL-6, 5 ng/ml IL-1β, 10 ng/ml IL-23, 3 μg/ml anti IL-4, 3 μg/ml anti IL-12, 3 μg/ml anti IFN-ɣ, and 3 μg/ml anti IL-2). Th17 Teffs were harvested and labeled with ^111^In-oxyquinoline and 20 × 10^6^^111^In-labeled Th17 Teffs were adoptively transferred to recipients treated with MPTP at dosages of 18 mg/kg every 2 hours for 4 doses. One hour prior to adoptive transfer and every 24 hours thereafter, recipients were treated ip with 10 mg/kg of either anti-MCAM or rat isotype control antibody. CT/SPECT images of each animal were acquired at 24, 48, and 72 hours post-transfer. Within tomographic images, electronic bit maps were drawn to circumscribe regions of interest that encompassed **(A)** brain, **(B)** lungs, **(C)** kidneys, **(D)** spleen, **(E)** cervical lymph nodes, **(F)** remaining lymph nodes, and included the entire body. Counts of radiolabeled T cells for each organ and entire body were determined by digital image analysis software (VIVID, GE Healthcare) and corrected for decay from the time of labeling. Counts for each organ were normalized as the percentage of total body counts at each time **(A-F)**. Means ± SEMs of radiolabel percentages were determined for 3–4 mice/treatment group and differences of percentages between isotype antibody and anti-MCAM treatment groups were determined by Student’s t-test where p ≤0.05 was considered significant.

To evaluate the role of MCAM and T cell migration into the CNS in the MPTP model, ^111^In-labeled donor Th17 Teffs were adoptively transferred to MPTP-intoxicated recipients that were treated with anti-MCAM or isotype control antibody. Mice were assessed by CT/SPECT at 24, 48, and 72 hours post–transfer. Immediately before transfer, 25% of donor T cells expressed MCAM as determined by flow cytometric analysis (data not shown). While no significant diminution of CD4+ cell migration was seen with the MCAM blocking antibody in the brain, lymph nodes, spleen, or kidneys, anti-MCAM treatment decreased accumulation of CD4+ cells in the lungs of MPTP mice (Figure 5). These data support the notion that Th17 Teffs are capable of extravasation and accumulating in the brains of MPTP mice when adequate numbers of Teffs are transferred. However, blocking MCAM did not adequately inhibit migration of donor T cells and may have enhanced accumulation, suggesting that MCAM may not play an active role in T cell extravasation within the SN in the MPTP model and may not play a role in exacerbation of neurodegeneration. The possibilities exist that very few Teffs, below the threshold of detection, are necessary to affect neurodegeneration or that Teffs can function at a distance in an endocrine or paracrine fashion without entering the brain parenchyma.

### Blocking MCAM provides partial neuroprotection in vivo

**Figure 6:**
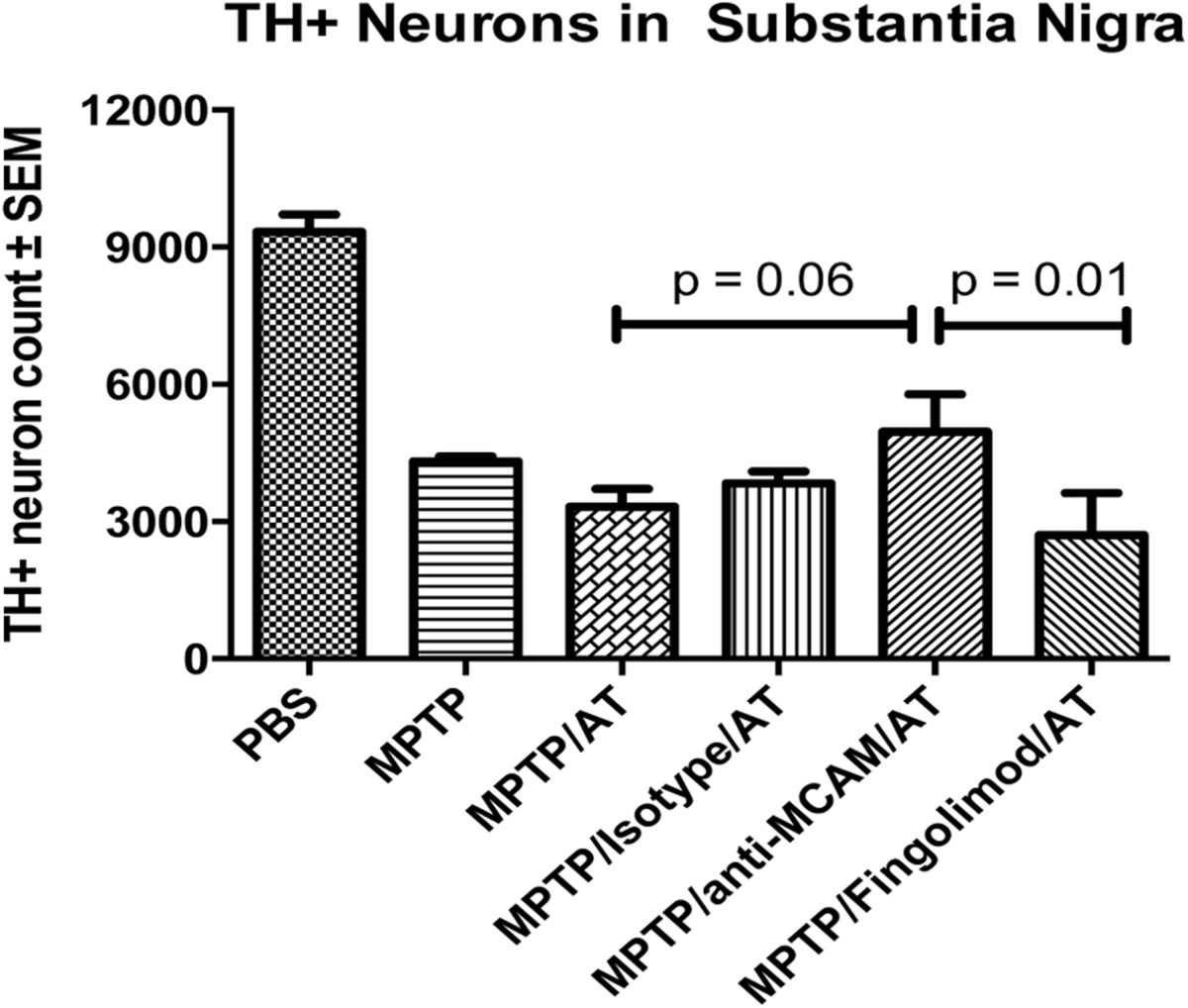
**Blocking MCAM following N-4YSyn-specific splenocyte transfers elicits partial neuroprotection.** Donor immune cells containing N-4YSyn-specific Teffs were obtained from spleens of mice immunized and boosted with N-4YSyn. To recipient mice that were treated with MPTP at dosages of 18 mg/kg every 2 hours for 4 doses, 30 × 10^6^ donor cells were adoptively transferred (AT) twelve hours after the last dose of MPTP, while one group of MPTP mice received no donor immune cells. Of the 4 groups that received donor immune cells, one group received no other treatment, one group was treated with 10 mg/kg rat isotype control antibody (MPTP/Isotype/AT), one group with 10 mg/kg anti-MCAM antibody (MPTP/anti-MCAM/AT), and one group with 1 mg/kg fingolimod (MPTP/fingolimod/AT). Antibody and fingolimod treatments began the day before adoptive transfer and continued until the end of study. One group was treated with only PBS (PBS), and served as total neuron control. Seven days after MPTP treatment, mice were terminally anesthetized, transcardially perfused with PBS for exsanguination, fixed with 4% paraformaldehyde in PBS, and brains removed and processed for immunohistochemistry. Brains were sectioned through the midbrain, immunostained with rabbit anti-tyrosine hydroxylase (TH) and HRP-conjugated goat anti rabbit IgG, and visualized with DAB. Total numbers of surviving dopaminergic neurons (TH+) in the SN were quantified by stereological analysis (Stereo Investigator, MBF Bioscience). Means ± SEMs of total numbers of surviving dopaminergic neurons were determined from 5–8 mice per treatment group and were compared by one way ANOVA and Fisher’s LSD post-hoc test.

To assess the role of MCAM in MPTP-induced neurodegeneration, the extent of Teff-mediated exacerbation of dopaminergic neuronal loss was measured in MPTP-treated mice. For these studies, N-4YSyn-specific Teffs were adoptively transferred to MPTP-treated recipient mice that were treated with anti-MCAM antibody, isotype control antibody, or fingolimod, a sphingosine-1 phosphate inhibitor that is known to sequester T cells within secondary lymphoid organs[157–160]. Seven days post-MPTP treatment, brains were removed, sections of midbrain stained for tyrosine hydroxylase (TH) expression, and surviving dopaminergic neurons determined by stereological analysis. Numbers of TH immunoreactive (TH+) neurons in SN from recipient mice treated with MPTP, Teffs, and anti-MCAM were slightly elevated, although not significantly (p = 0.06) compared to those treated with MPTP and Teffs that received either isotype control antibody or no other treatment (Figure 6). This suggested that MCAM may not play a critical role in Teff-mediated exacerbation of dopaminergic neurodegeneration, but rather may have a minor effect on neurodegenerative processes if blocked. Interestingly, numbers of TH+ neurons from animals treated with MPTP, Teffs, and fingolimod were significantly diminished compared to those similarly treated recipients that receive anti-MCAM. This indicated that blockage of sphingosine-1-phosphate receptor and sequestration of T cells significantly increased the neurodegenerative processes. This may be due to the inability of fingolimod to sequester effector memory T cells from circulation[159–161] or that Tregs may be preferentially sequestered in lymphoid tissues with loss of their neuroprotective capability in the brain[162]. Moreover, these data do not rule out the possibility that Teffs in the MPTP model may function outside the brain parenchyma.

## Conclusions

Considerable evidence supports the notion that T cell-mediated immunity plays an important role in disease progression in neurodegenerative disorders. This is most evident in MS with increased activity of autoreactive Teffs for self-antigens that comprise the myelin sheath. MS seems to be the most extreme disorder whereby unregulated T cell immunity, for the most part, mediates direct neurotoxicity as well as drives other components of the immune system to produce autoantibodies (B cells) and neuroinflammation (reactive microglia and infiltrating macrophages). Expectedly in MS, Treg levels and function are most notably found to be diminished compared to the unaffected populations. In ALS, numerous alterations in adaptive immunity are also well-known, and range from increased levels of anti- self-antibodies to diminished Treg levels and increased levels of proinflammatory Teffs not only in the peripheral blood, but also in sites of neurodegeneration within the spinal cords of ALS patients. Moreover, increased expression of MHC II by APCs and microglia/macrophages suggests the association of a systemically activated immune system in ALS. AD patients also exhibit significant aberrations in immune cells and function. Neurodegeneration in AD is also thought to be associated with increased neuroinflammation that drives disease progression. While wide variability in levels of immune cells is exhibited among studies of AD patients, CD4+ T cells are chiefly reported to be in fluctuation with opposing pro-inflammatory Teffs and anti-inflammatory T cells or T cell functions at imbalance. The recent clinical trial of an Aβ1-42 experimental vaccine in AD patients which drove immune responses of some patients toward a T cell-mediated meningoencephalitis underscores the putative precipice by which this imbalance hangs and awaits only the slight nudge of the T cell response to drive disease progression toward either neurotoxicity or neuroprotection. In PD, loss of dopaminergic neurons is also associated with a neuroinflammatory component, which is thought to play a key role in disease progression. Recent studies showed that PD patients exhibit increased levels of T cells with effector/memory phenotypes as well as corresponding diminution of Treg function, both correlating with the severity of motor dysfunction. Taken together, the preponderance of evidence supports a role for adaptive immune responses in neurodegenerative disorders, particularly those associated with a neuroinflammatory component. However, the mechanism(s) utilized and the epitopes recognized have yet to be determined for each of these disorders. One perspective is that with low regulatory function, peripheral Teffs, which are normally kept in check, can expand and evade control to exacerbate disease processes and accelerate or amplify disease progression. In different proteinopathies, the possibility exists that the modified, misfolded, and aggregated proteins associated with each particular disorder (MBP, MOG, SOD1, Aβ, tau, or α-syn) are not adequately degraded or eliminated, and eventually drain with inflammatory mediators to peripheral lymphoid tissues, wherein they are preferentially processed and presented by APCs. Proinflammatory environments in the draining tissues with increased presentation of modified self-proteins could provide conditions more conducive to elicitation of Teffs such as Th1 and Th17 cells. Marshaling those Teffs to sites of neuroinflammation, and evoking their destruction potential would provide added neurotoxic conditions. Conversely, mobilizing anti-inflammatory T cells, such as Th2 and Tregs, to these same sites may provide neuroprotective responses and serve as therapeutic strategies to control the proinflammatory processes of neurodegeneration. Thus, the mechanism(s) of T cell migration and extravasation to sites of neuroinflammation remain major issues in neurodegenerative disorders. Indeed, with the exception of MS whereby treatment with fingolimod or natalizumab limits T cell migration and extravasation, the necessity of Teff infiltration for exacerbation of neurotoxicity in other neurodegenerative disorders has not been established.

The lack of significant neuroprotection afforded with the administration of anti-MCAM described herein could be due to a number of factors. First, administration of the MCAM blocking antibody may have been needed at times other than 1 hour prior to adoptive transfer or at a greater concentration to ensure that the antibody had sufficient opportunities to bind to the MCAM receptor. Second, the relatively low percentage of adoptively transferred cells that expressed MCAM prior to adoptive transfer could have proven problematic since the antibody could not have been able to find its target within the entire circulation. Third, the use of N-4YSyn as stimulation instead of anti-CD3 may not have activated the cells enough to increase infiltration into the CNS. The amount of N-4YSyn specific cells that were transferred into MPTP-intoxicated MPTP mice may have been an insufficient number to see increased migration leading to a lack of significant differences between MPTP and PBS treated mice as well. Lastly, T cell extravasation from the circulation does not rely on one CAM alone, but rather is a combination of CAMs that work in a redundant and synergistic fashion to allow cells to cross endothelial cell barriers. This leads to the possibility that blocking MCAM only is not sufficient to halt T cell infiltration as other CAMs could take over the role of MCAM. If complete and significant blockage of CD4+ cell entry to the CNS is the ultimate goal, administration of a variety of CAM blocking agents may be necessary to target multiple steps in immune cell extravasation.

## Electronic supplementary material

Additional file 1: **CT/SPECT imaging of migration and accumulation of**^**111**^**In-labeled Teffs in PBS-treated mice.** CD3+ T cells were harvested and enriched from spleens and lymph nodes of C57BL/6 donor mice. Isolated cells were activated using anti-CD3 for 3 days. Recipient mice were treated with 4 doses of PBS; one dose every 2 hours. Twelve hours after the last injection, activated Teffs were labeled with ^111^In-oxyquinoline and 20 × 10^6^ labeled cells were adoptively transferred into PBS-treated recipient mice. CT/SPECT images were acquired at 24 hours after transfer. (ZIP 1 MB)

Additional file 2: **CT/SPECT imaging of migration and accumulation of**^**111**^**In-labeled Teffs in MPTP-treated mice.** CD3+ T cells were harvested and enriched from spleens and lymph nodes of C57BL/6 donor mice. For 3 days, isolated cells were activated using anti-CD3. Recipient mice were treated with 4 doses, one dose every 2 hours, of MPTP at 18 mg/kg. Twelve hours post MPTP injection, activated Teffs were labeled with ^111^In-oxyquinoline and 20 × 10^6^ labeled cells were adoptively transferred into MPTP-treated recipient mice. CT/SPECT images were acquired at 24 hours after transfer. (ZIP 930 KB)

## Abbreviations

^111^In: ^111^Indium
α-syn: Alpha-synuclein
AD: Alzheimer’s disease
ALS: Amyotrophic lateral sclerosis
APC: Antigen presenting cell
Aβ: Amyloid beta
AT: adoptive transfer
BBB: Blood brain barrier
CAM: Cell adhesion molecule
CLT: Cytotoxic T lymphocyte
CNS: Central nervous system
Cop-1: Copolymer-1
CSF: Cerebral spinal fluid
CT: Computed tomography
DAMP: Damage-associated molecular patterns
EAE: Experimental autoimmune encephalomyelitis
EC: Endothelial cell
FasL: Fas ligand
FoxP3: Forkhead box P3
GM-CSF: Granulocyte macrophage colony stimulating factor
ICAM-1: Intercellular adhesion molecule 1
Ig: Immunoglobulin
IPEX: Immunodysregulation polyendocrinopathy enteropathy X-linked
iTreg: Induced regulatory T cell
LB: Lewy body
LN: Lewy neurite
LPS: Lipopolysaccharide
MAMP: Microorganism-associated molecular patterns
MBP: Myelin basic protein
MCAM: Melanoma cell adhesion molecule
MHC: Major histocompatibility complex
MPTP: 1-methyl-4-phenyl-1,2,3,6-tetrahydropyridine
MS: Multiple sclerosis
N-α-syn: Nitrated-α-syn
N-4YSyn: Nitrated C-terminal peptide of N-α-syn
nTreg: Natural regulatory T cell
PBS: Phosphate buffered saline
PD: Parkinson’s disease
PECAM-1: Platelet endothelial cell adhesion molecule 1
PNS: Peripheral nervous system
PRR: Pattern recognition receptor
RNS: Reactive nitrogen species
ROS: Reactive oxygen species
SN: Substantia nigra
SPECT: Single photon emission CT
TCR: T cell receptor
Teff: Effector T cell
Th: Helper T cell
Treg: Regulatory T cell
UPDRS III: Unified Parkinson’s Disease Rating Scale Part III
VCAM-1: Vascular cell adhesion molecule 1
VIP: Vasoactive intestinal peptide
WT: Wild type.
